# Modeling Microplastic Transport in the Marine Environment: Testing Empirical Models of Particle Terminal Sinking Velocity for Irregularly Shaped Particles

**DOI:** 10.1021/acsestwater.2c00466

**Published:** 2023-03-22

**Authors:** Róisín Coyle, Matthew Service, Ursula Witte, Gary Hardiman, Jennifer McKinley

**Affiliations:** † Civil Engineering, School of Natural and Built Environment, Queen’s University Belfast, Belfast BT7 1NN, Northern Ireland, U.K.; ‡ Agri-Food and Biosciences Institute, 18a Newforge Lane, Belfast BT9 5PX, Northern Ireland, U.K.; § School of Biological Sciences, University of Aberdeen, Aberdeen AB24 3FX, U.K.; ∥ School of Biological Sciences, Institute for Global Food Security (IGFS), Queen’s University Belfast, 19 Chlorine Gardens, Belfast BT9 5DL, Northern Ireland, U.K.; ⊥ Department of Medicine, Medical University of South Carolina, Charleston, South Carolina 29425, United States; # Geography, School of Natural and Built Environment, Queen’s University Belfast, Belfast BT7 1NN, Northern Ireland, U.K.

**Keywords:** microplastics, transport modeling, settling velocity, drag coefficient, irregular particles, microplastic vertical transport

## Abstract

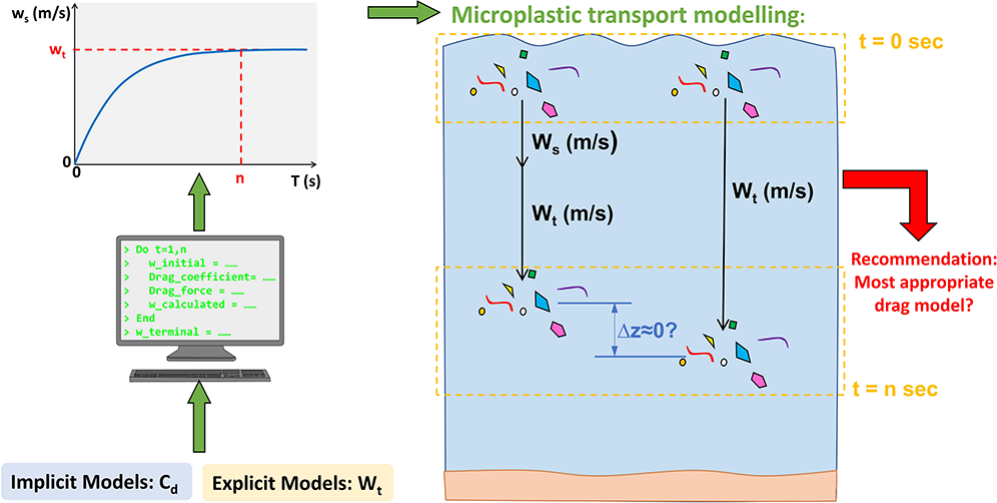

Microplastic (mP) pollution has been indicated as an area of concern in the marine environment. However, there is no consensus on their potential to cause significant ecological harm, and a comprehensive risk assessment of mP pollution is unattainable due to gaps in our understanding of their transport, uptake, and exchange processes. This research considers drag models that have been proposed to calculate the terminal settling velocity of regularly and irregularly shaped particles to assess their applicability in a mP modeling context. The evaluation indicates three models that predict the settling velocity of mPs to a high precision and suggests that an explicit model is the most appropriate for implementation in a mP transport model. This research demonstrates that the mP settling velocity does not vary significantly over time and depth relevant to the scale of an ocean model and that the terminal settling velocity is independent of the initial particle velocity. These findings contribute toward efforts to simulate the vertical transport of mPs in the ocean, which will improve our understanding of the residence time of mPs in the water column and subsequently their availability for uptake into the marine ecosystem.

## Abstract 2

Settling velocity models can be implemented in microplastic ocean transport models for irregularly shaped particles with unknown initial settling velocity.

## 1 Introduction

Plastic is the overwhelmingly predominant type of litter in the environment,^1^ with particle transport models suggesting that up to 51 trillion plastic particles are floating on the ocean surface.^2^ Plastic particles that are less than 5 mm in size are known as microplastics (mPs) and are estimated to account for 92% of floating plastic particles in the ocean worldwide.^3^ They are difficult to remove from natural water streams and persist in the marine environment for long time periods, breaking into continually smaller particles through slow degradation processes that may take hundreds of years.^4,5^

The determination of the ecological harm caused by mPs in the marine environment is a key objective within the EU Marine Strategy Framework Directive (MSFD 2008/56/EC).^6,7^ However, uncertainties surrounding mP processes and a lack of consensus on their fate in the oceans, including their potential to bioaccumulate within ecosystems, prohibit a comprehensive risk assessment of mP pollution.^8^ Understanding the physical transport of mPs is a key step toward elucidating their fate and impacts in the marine environment. The determination of the sinking rate of mPs is essential to modeling their vertical transport since it impacts their residence time in the water column, which in turn influences their fate and bioavailability.

Numerous models have been developed to calculate the terminal settling velocity of natural particles and, more recently, consider the wider range of morphologies exhibited by mPs, including fibers. The study by Van Melkebeke et al.^9^ evaluated 11 shape-dependent drag models to calculate the settling velocity of irregularly shaped mPs and concluded that Dioguardi et al.’s model^10^ was the most accurate based on its low average error. However, an erroneous version of the model by Bagheri and Bonadonna^11^ was used to reach this conclusion. It has since been suggested that the model by Zhang and Choi^12^ is more accurate in predicting the settling velocity of fibers. Therefore, it would be beneficial to confirm that Van Melkebeke et al.’s conclusion is still valid when the new and revised models are considered.

When comparing explicit models, the model by Francalanci et al.^13^ was found to be more accurate in predicting mP settling velocity than the model by Dietrich,^14^ particularly for irregular particles with low Corey shape factor (CSF) values. The newly proposed model by Yu et al.^15^ has since been found to predict the settling velocity of mPs with lower error than Francalanci et al.’s^13^ model. However, no direct comparison has been made between this model and Dioguardi et al.’s model.^10^

Thus, this research paper evaluates the six drag models mentioned above and a reference model for spheres^16^ to make a more complete comparison of their performance in predicting the terminal settling velocity of mPs and to reach a conclusion on which model is most appropriate in a mP modeling context. A description of each of the empirical models evaluated is included in Supporting Information 1.

This evaluation reported here considers only the vertical transport of mPs under the action of gravitational, buoyant, and drag forces in a quiescent water column. Additional processes that influence the vertical transport of mPs including wind-mixing,^17^ weather events,^18^ biofouling,^19^ and incorporation into aggregates^20^ and fecal pellets^21^ are not considered.

## 2 Methods and Data

### 2.1 Approach

The approach adopted in this study followed a series of stages.

1. The seven models selected were evaluated using the dataset in Van Melkebeke et al.^9^ This dataset is described in Section 2.4 and summarized in Table 1.
2. Yu et al.’s model^15^ was re-evaluated using the dataset from Dioguardi et al.^10^ This dataset is described in Section 2.4 and summarized in Table 1.
3. The impact of the choice of initial velocity on the result of the implicit models was investigated.
4. The variation in the terminal settling velocity over the range of density in the ocean was explored to test the impact of assuming a constant settling velocity in a mP transport model.
5. The impact of using a constant sinking velocity on the distance traveled by the mPs was explored.

**Table 1:** Outline of Experimental Datasets Used to Complete the Model Evaluation in This Study

Dataset used:	Van Melkebeke et al.	Dioguardi et al.
**variables included:**	measured terminal settling velocity (*w*_meas_)	measured terminal settling velocity (w_meas_)
	fluid dynamic viscosity (μ_f_)	fluid dynamic viscosity (μ_f_)
	fluid density (ρ_f_)	fluid density (ρ_f_)
	volume equivalent sphere diameter (d_p_)	volume equivalent sphere diameter (d_p_)
	particle density (ρ_p_)	particle density (ρ_p_)
	longest, intermediate, and shortest particle dimensions (a, b, and c)	longest, intermediate, and shortest particle dimensions (a, b, and c)
	sphericity (Φ)	sphericity (Φ)
	Dellino shape factor (Ψ)	Dellino shape factor (Ψ)
	circularity (χ)	circularity (χ)
	powers roundness index (*P*)	maximum projection area (*A*_mp_)
	particle shape category	maximum projection perimeter (*P*_mp_)
	fluid kinematic viscosity (ν_f_)	
**method of measuring terminal settling velocity:**	**traditional cylindrical settling column experiments with following setup:**	**traditional cylindrical settling column experiments with following set up:**
	**settling column:** 45 cm height and 10 cm diameter	**settling column:** 150 cm height and 5 cm inner radius
	**fluid used:** deionized water or ethanol (depending on particle density)	**fluid used:** two glycerin solutions
	**time recording:** time taken to travel two times 10 cm using a high dynamic range camera at 100 frames/sec	**settling velocity recording:** using a high-definition video camera at 50 frames/sec
**method of characterizing particle shape:**	**Particle size:** sieve shaker	**Grain size:**combination of sieving and particle counting techniques
	**Shape parameters:** High-resolution images generated using a digital microscope and analyzed using image analysis software ImageJ.	**Shape parameters:** Image analysis techniques on high-resolution photographs under a stereomicroscope.
**rationale for using the dataset:**	Dataset contains all the detailed particle shape information required to implement each of the models under evaluation	Dataset contains all the detailed particle shape information required to independently evaluate the performance of Yu et al.^15^
**reference for full experimental details:**	(9)	(10)

### 2.2 Method to Evaluate Explicit Models

Empirical models that provide an expression to directly calculate the terminal settling velocity of particles falling in a fluid are known as explicit models. These models are computationally more efficient than implicit models since they do not require an iterative calculation. For each of the explicit models tested, the particle properties from the dataset were directly substituted into the models to output a single value of the terminal sinking velocity. The specific procedure to implement each of the explicit models is outlined in Supporting Information 6–8.

### 2.3 Method to Evaluate Implicit Models

Implicit models give an expression for the drag coefficient *C*_D_ and require an iterative method to calculate the terminal settling velocity. The iterative method outlined in Dioguardi et al.^10^ and Bagheri and Bonadonna^11^ was applied to test the implicit models. First, the particle Reynolds number *Re* was calculated using an assumed initial settling velocity and the particle properties in the dataset. The drag coefficient *C*_d_ was calculated using *Re* and subsequently utilized to calculate the drag force. The gravitational and buoyant forces acting on the particle were calculated using the particle properties and used alongside the drag force to estimate the net vertical force acting on the particle. The net force was substituted into the equation ∑*F* =  to calculate the settling velocity at the next time step. The particle Reynolds number *Re* was then recalculated, and the process was restarted. The iterations continued until the drag force equaled the sum of the gravitational and buoyant forces, and the particle acceleration became negligible (less than 0.001 m/s^2^). At this point, it was assumed that the terminal settling velocity was attained. The specific procedure to implement each of the implicit models tested is outlined in Supporting Information 2–5.

This method differs from the method of model evaluation used in Van Melkebeke et al.,^9^ where the terminal settling velocity was already known and was used as the initial assumed velocity. The drag coefficient *C*_d_ was obtained in the same manner as outlined above but was then implemented into Newton’s impact formula^22^ to calculate the terminal settling velocity. This was compared to the measured terminal settling velocity to evaluate the model performance. In a mP transport modeling context, when the terminal settling velocity of the particle is unknown, it is more useful to apply implicit models using the iterative method wherein the initial velocity is assumed, and the calculation is iterated until the terminal settling velocity is attained.

### 2.4 Datasets Used

The dataset in Van Melkebeke et al.^9^ was used to evaluate the models under consideration. This dataset contains information on the terminal settling velocity of 140 mPs that was obtained during cylindrical settling column experiments. A summary of the experimental methods is included in Table 1, and full experimental details can be found in their paper.^9^ The mPs were generated from a range of product types to encompass the array of regular and irregular particle morphologies exhibited by mPs, including three-dimensional (3D) shapes such as granules, spheres, and fragments; quasi-two-dimensional (2D) shapes such as films; and quasi-one-dimensional (1D) shapes such as fibers and lines. The particles were quantified into 3 shape categories using image analysis software and microscopy, and, in total, the dataset contains 80 fragments, 40 films, and 20 fibers. A variety of shape descriptors were also estimated, but sphericity was found to be the only descriptor that could adequately distinguish between the three distinct morphologies. This comprehensive dataset was used to evaluate the models under consideration as it contained all the detailed information on particle morphology required to successfully implement the models.

This dataset was used to fit the models by Yu et al.^15^ and Zhang and Choi^12^ and, we noted, could falsely overrepresent their performance. For example, Yu et al.’s model^15^ revealed high-performance characteristics when using this dataset and thus warranted further testing using an independent dataset.

As Yu et al.’s model was fitted using all the data currently available on mP settling velocity, the dataset compiled for volcanic ash particles in Dioguardi and Mele^23^ and revised in Dioguardi et al.^10^ was instead used to retest the models. This dataset is similar to Van Melkebeke et al.’s^9^ dataset in that it contains information on the terminal settling velocity of irregularly shaped particles obtained during cylindrical settling column experiments. It also contains detailed information on each particle’s morphology, which is required to successfully implement the models but was not used to fit either of the models highlighted above, making it an appropriate choice to retest Yu et al.’s^15^ model. A summary of the experimental methods used to obtain this dataset is outlined in Table 1, and full experimental details can be found in the relevant paper.^10,23^

### 2.5 Analysis Undertaken during Model Evaluation

Several indicators of the model’s ability to reproduce the measured terminal velocity were used during the model evaluation.

Linear regression was used to fit a model in the form *y* = *mx* to understand the relationship between the calculated settling velocity (*y*) and the measured settling velocity (*x*). In this instance, an m-value close to 1 indicates that the model accurately predicts the terminal settling velocity of the particles, with *m* < 1 suggesting that the model underestimates the terminal settling velocity and *m* > 1 suggesting that the model overestimates the terminal settling velocity. The coefficient of determination *r*^2^ indicates the amount of variability in the estimated velocity that can be explained by the linear regression model, with an *r*^2^ value close to 1 suggesting that it adequately fits the data and can explain the variability in the estimated terminal settling velocity.

The average absolute relative error (|AE|) measures the difference between the calculated and measured velocity as a percentage of the measured velocity
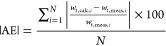
where *N* is the number of data points. For an individual particle, the absolute relative error is zero when the measured and calculated velocity are equal. An absolute relative error of 50% for an individual particle indicates that the calculated velocity is either 50% smaller or 50% larger than the measured velocity. The overall average absolute relative error therefore will demonstrate the difference between the modeled terminal settling velocity and the measured terminal settling velocity but will not provide an indication of whether the model overestimates or underestimates the particle terminal settling velocity.

The root-mean-square error (RMSE) is the square root of the average of the squared error and provides an absolute measure of the model’s ability to predict the measured terminal settling velocity. For consistency, we calculate this statistic using the same expression as Van Melkebeke et al.^9^ and Dioguardi et al.^10^
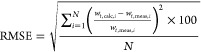
where *N* is the number of data points. By squaring the relative error, RMSE applies more weight to large errors, and a low RMSE indicates that the model accurately predicts the settling velocity.

## 3 Results and Discussion

### 3.1 Model Evaluation

**Figure 1:**
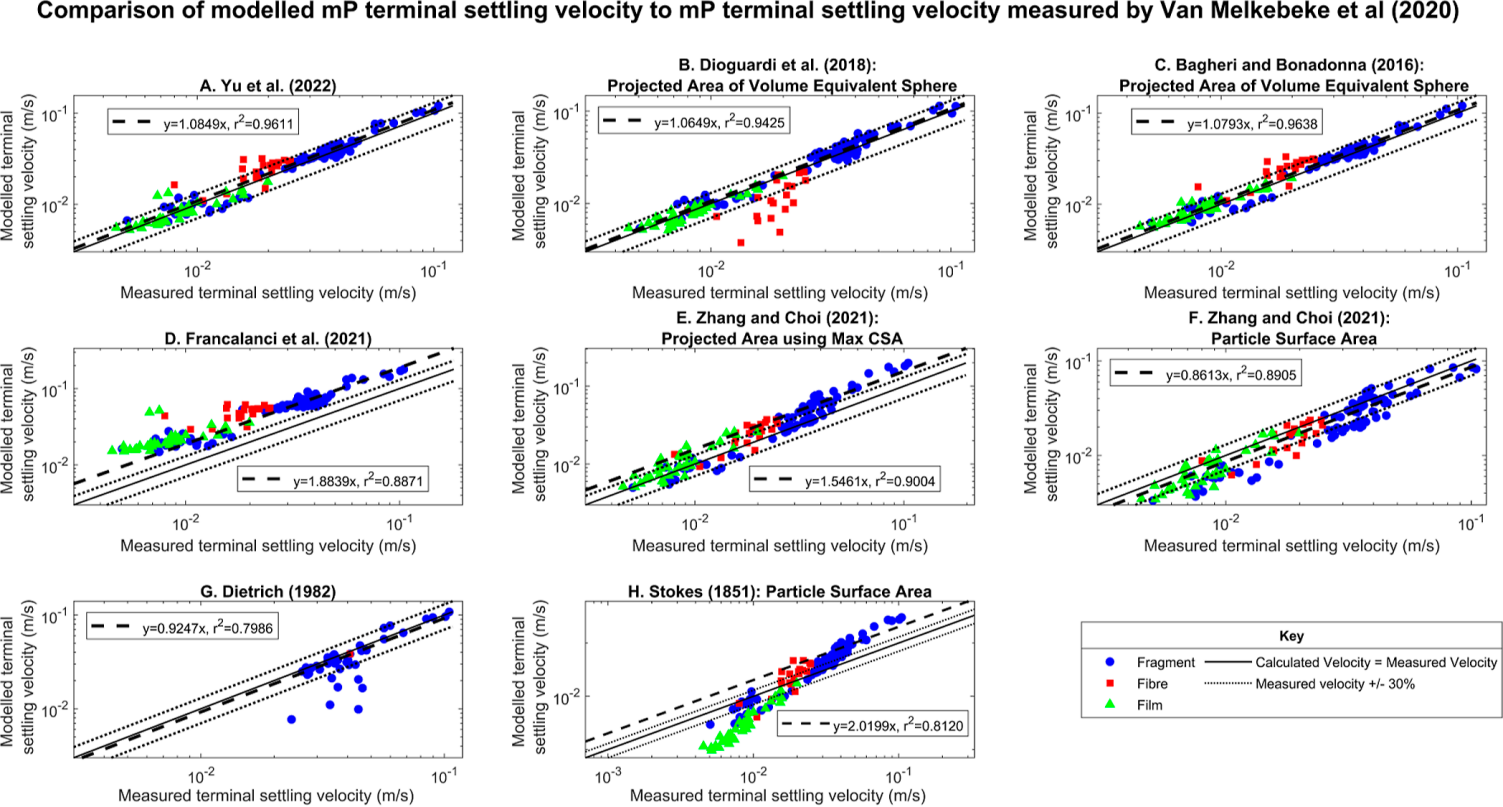
Output for each model evaluated showing the model-estimated terminal settling velocity against the measured terminal settling velocity from the dataset in Van Melkebeke et al*.*^9^ The solid line indicates the ideal fit where the estimated terminal settling velocity equals the measured terminal settling velocity, and the dotted lines indicate the estimated terminal settling velocity equals ±30% of the measured terminal settling velocity. The dashed line indicates the best fit line in the form *y* = *mx* that was obtained using linear regression. The labels A–H distinguish between the results of the models evaluated. (A) Yu et al.’s model.^15^ (B) Dioguardi et al.’s model^10^ using the particle projection area as the particle effective area. (C) Bagheri and Bonadonna’s model^11^ using the particle projection area as the particle effective area. (D) Francalanci et al’s model.^13^ (E) Zhang and Choi’s model^12^ using the maximum cross-sectional area as the particle effective area. (F) Zhang and Choi’s model^12^ using the particle surface area as the particle effective area. (G) Dietrich’s model.^14^ (H) Stokes model^16^ using the particle surface area as the particle effective area.

Considering all the mP morphologies, Dioguardi et al.’s model^10^ produces the lowest m value of 1.06 (*r*^2^ = 0.94), suggesting that it accurately predicts the terminal settling velocity of the mP particles in the dataset. The models by Bagheri and Bonadonna^11^ and Yu et al.^15^ both produce a similar m value of 1.08. However, Dioguardi et al.’s^10^ model underestimates the settling velocity of fibrous particles (Figure 1), and as a result, it has a higher absolute average relative error (|AE| = 15.82%) than both Bagheri and Bonadonna^11^ (|AE| = 13.95%) and Yu et al.^15^ (|AE| = 14.81%) (Table 3). It should be noted that these errors are generated relative to the measured terminal settling velocity. Furthermore, they are obtained using distinct models with *m*-value and *r*^2^ metrics which provide a confidence measure in the data. These models also have the highest overall coefficient of determination (*r*^2^ = 0.96) (Table 2), indicating that the linear regression model *y* = *mx* is a better fit to their output.

**Table 2:** Summary of Output from Regression Analysis Undertaken during Model Evaluation

	overall		fragments only		films only		fibers only	
model	*m*	*r*^2^	*m*	*r*^2^	*m*	*r*^2^	*m*	*r*^2^
Dioguardi et al. (2018)^a^	1.06	0.94	1.09	0.95	0.97	0.94	0.66	0.40
Bagheri and Bonadonna (2016)^a^	1.08	0.96	1.07	0.97	1.05	0.90	1.30	0.58
Yu et al. (2022)	1.08	0.96	1.08	0.96	0.95	0.71	1.27	0.47
Dietrich (1982)	0.92	0.80	N/A	N/A	N/A	N/A	N/A	N/A
Zhang and Choi (2021)^b^	0.86	0.89	0.86	0.86	0.88	0.79	0.87	0.60
Zhang and Choi (2021)^c^	1.55	0.90	1.56	0.88	1.31	0.79	1.37	0.64
Francalanci et al. (2021)	1.88	0.89	1.83	0.93	2.38	–0.06	2.58	–0.02
Stokes (1851)^b^	2.02	0.81	2.10	0.80	0.49	0.64	1.42	0.51

a Indicates that the projected area of the volume equivalent sphere was used as the effective area in the calculation of the drag force.

b Indicates that the particle surface area was used as the effective area in the calculation of the drag force and.

c Indicates that the maximum cross-sectional area was used as the effective area in the calculation of the drag force.

The *m*-value of the regression model fitted to the output using Dietrich’s model^14^ (*m* = 0.92) (Table 2) deviated from 1 to the same degree as Bagheri and Bonadonna^11^ and Yu et al.’s^15^ model. However, Dietrich’s^14^ model produced a lower *r*^2^ value (0.80) (Figure 1) and a higher |AE| (|AE| = 19.43%) (Table 3) when all particles were considered, indicating that it is less accurate in reproducing the measured terminal settling velocity, and the regression model is less appropriate in explaining the variation in the calculated terminal settling velocity. This is evident in Figure 1. Furthermore, as Dietrich’s model^14^ is not recommended for use when CSF < 0.2 and is invalid when CSF < 0.15, it was only applicable to one fibrous particle and none of the film particles. Therefore, this model will not be useful in a modeling context for irregularly shaped mPs.

**Table 3:** Summary of Errors in the Estimated Terminal Settling Velocity for Each Model Evaluated Compared to the Measured Terminal Settling Velocity for All Particles from the Dataset by Van Melkebeke et al.^9^^,^ a

	overall		
model	AE	|AE|	RMSE
Bagheri and Bonadonna (2016)^b^	8.97	13.95	20.56
Yu et al. (2022)	6.21	14.81	22.67
Dioguardi et al. (2018)^b^	–1.47	15.82	21.28
Dietrich (1982)	–14.70	19.43	28.46
Zhang and Choi (2021)^c^	–18.60	23.48	27.75
Zhang and Choi (2021)^d^	28.44	33.80	43.81
Stokes (1851)^c^	11.18	59.88	73.43
Francalanci et al. (2021)	128.31	128.31	151.07

a AE = Average relative error (%), |AE| = average absolute relative error (%), and RMSE = root-mean-square error (%).

b Indicates that the projected area of the volume equivalent sphere was used as the effective area in the calculation of drag force.

c Indicates that the particle surface area was used as the effective area in the calculation of drag force.

d Indicates that the maximum cross-sectional area was used as the effective area in the calculation of drag force.

**Figure 2:**
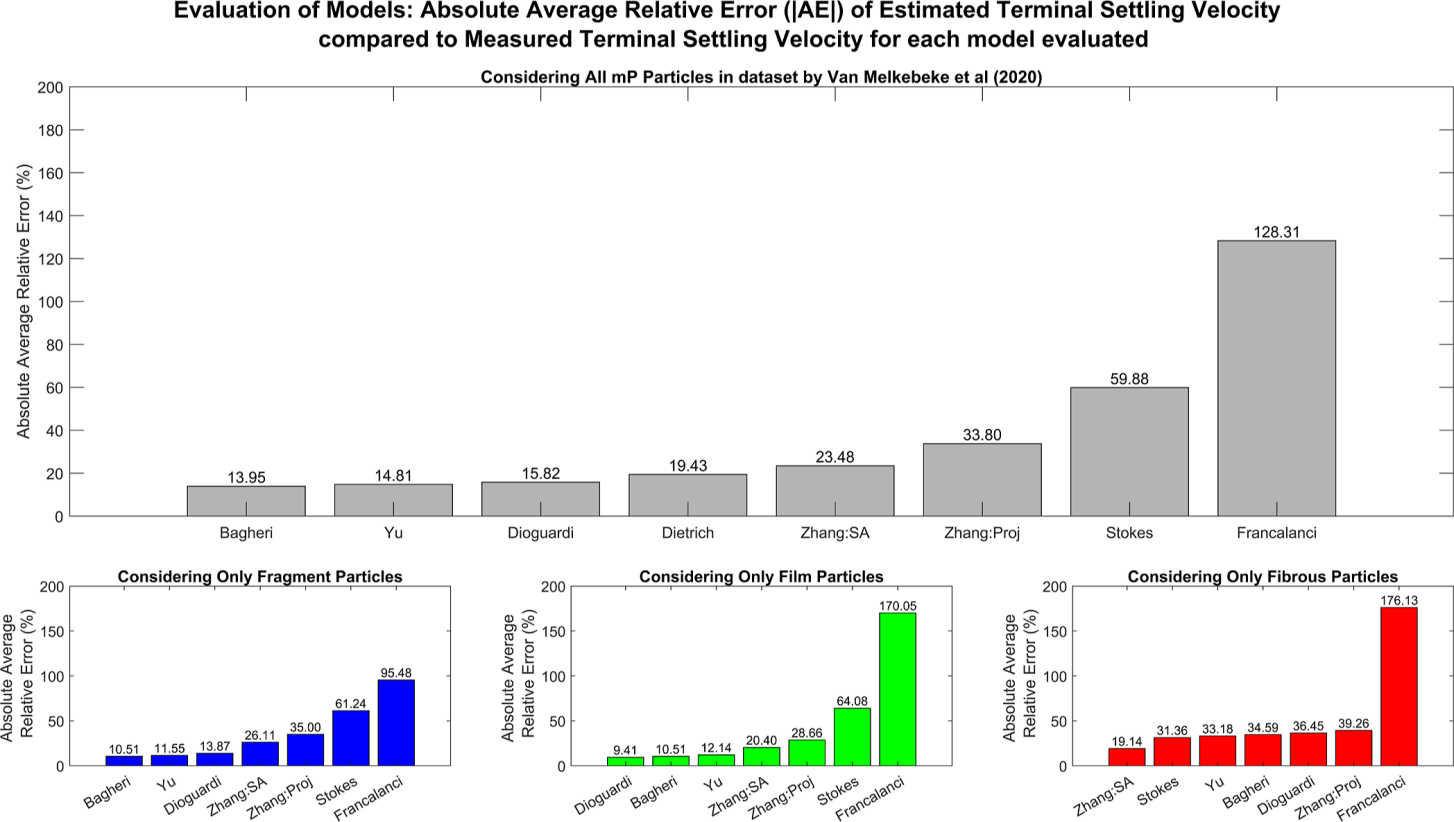
Absolute average relative error of model-estimated terminal settling velocity for each model evaluated compared to the measured terminal settling velocity from the dataset by Van Melkebeke et al..^9^ The main figure illustrates the absolute average relative error for the entire dataset, while the lower figure shows the error when each morphology within the dataset is considered separately. The key for the models evaluated is Stokes = Stokes model^16^ using the particle surface area as the particle effective area, Bagheri = Bagheri and Bonadonna’s model^11^ using the particle projection area as the particle effective area, Dioguardi = Dioguardi et al.’s model^10^ using the particle projection area as the particle effective area, Zhang:SA = Zhang and Choi’s model^12^ using the particle surface area as the particle effective area, Zhang:Proj = Zhang and Choi’s model^12^ using the maximum cross-sectional area as the particle effective area, Dietrich = Dietrich’s model,^14^ Francalanci = Francalanci’s model,^13^ and Yu = Yu et al.’s model.^15^

The model by Zhang and Choi^12^ provides the next best estimate of the measured terminal settling velocity when all morphologies are considered. The output of this model is improved when the particle surface area is used as the effective area (|AE| = 23.48%) in the drag force calculation rather than using the projection area as outlined in the paper (|AE| = 33.80%) (Figure 2).

The models by Francalanci et al.^13^ and Stokes’^16^ are less accurate, and their calculated terminal settling velocity deviates from the measured terminal settling velocity by an average of 128.31 and 59.88%, respectively, when all morphologies are considered (Table 3). As Stokes’ model^16^ is a reference law for spherical particles and irregularly shaped particles in the dataset experience more drag, it is expected that it will overestimate their settling velocity. Francalanci et al.’s model^13^ overestimates the terminal settling velocity of all morphologies considered, with an overall |AE| of 95.48, 170.05, and 176.13% for fragments, films, and fibers, respectively (Table 4). Similar results were obtained when Francalanci et al.^13^ validated their model using the same dataset.

**Table 4:** Summary of Errors in the Estimated Terminal Settling Velocity for Each Model Evaluated Compared to the Measured Terminal Settling Velocity for mP Particles in the Dataset by Van Melkebeke et al.^9^ Separated by Particle Morphology^a^

	fragments only			films only			fibers only		
model	AE	|AE|	RMSE	AE	|AE|	RMSE	AE	|AE|	RMSE
Bagheri and Bonadonna (2016)^b^	3.21	10.51	13.27	8.60	10.51	15.02	32.75	34.59	42.47
Yu et al. (2022)	3.54	11.55	14.95	–0.68	12.14	20.42	30.63	33.18	43.23
Dioguardi et al. (2018)^b^	7.27	13.87	16.69	–2.07	9.41	11.05	–35.23	36.45	42.56
Dietrich (1982)	N/A	N/A	N/A	N/A	N/A	N/A	N/A	N/A	N/A
Zhang and Choi (2021)^c^	–21.13	26.11	30.20	–15.68	20.40	24.22	–14.33	19.14	23.89
Zhang and Choi (2021)^d^	28.42	35.00	46.49	25.24	28.66	37.16	34.90	39.26	45.06
Stokes (1851)^c^	43.78	61.24	79.87	–64.08	64.08	66.25	31.30	31.36	58.75
Francalanci et al. (2021)	95.48	95.48	102.56	170.05	170.05	200.36	176.13	176.13	193.36

a AE = average relative error (%), |AE| = average absolute relative error (%), RMSE = root-mean-square error (%).

b Indicates that the projected area of the volume equivalent sphere was used as the effective area in the calculation of drag force.

c Indicates that the particle surface area was used as the effective area in the calculation of drag force.

d Indicates that the maximum cross-sectional area was used as the effective area in the calculation of drag force.

The terminal settling velocity of the fragment particles is most accurately reproduced using Bagheri and Bonadonna’s model,^11^ which has an m value of 1.07, an *r*^2^ value of 0.97 (Table 2), and the lowest |AE| at 10.51% (Table 4). This occurred since the model was derived using mainly volcanic particles and ellipsoids which are analogous in morphology to fragment mPs. Comparable results are obtained for fragments when Yu et al.’s explicit model^15^ is used, with an m value of 1.08, an *r*^2^ value of 0.96 (Table 2), and an |AE| of 11.55% (Table 4) and also when Dioguardi et al.’s implicit model^10^ is used, with an m value of 1.09, an *r*^2^ value of 0.95 (Table 2), and an |AE| of 13.87% (Table 4).

Dioguardi et al.’s model^10^ provides the closest estimate of the measured terminal settling velocity of film particles, with an *m* value of 0.97, an *r*^2^ value of 0.94 (Table 2), and an |AE| of 9.41% (Table 4). Bagheri and Bonadonna^11^ and Yu et al.’s^15^ model both provide similarly accurate results with m values of 1.05 and 0.95, respectively (Table 2). However, Yu et al.’s model^15^ produces a lower coefficient of determination (*r*^2^ = 0.71) (Table 2) and higher |AE| (|AE| = 12.14%) than Bagheri and Bonadonna’s model^11^ (*r*^2^ = 0.90 and |AE| = 10.51%) (Tables 2 and 4), indicating that it is less accurate in reproducing the terminal settling velocity of films. This is unexpected since film mPs were included in the derivation of Yu et al.’s model,^15^ and so it should provide a better prediction of their terminal settling velocity than the models by Bagheri and Bonadonna^11^ and Dioguardi et al.^10^ which did not take into account film particles.

**Figure 3:**
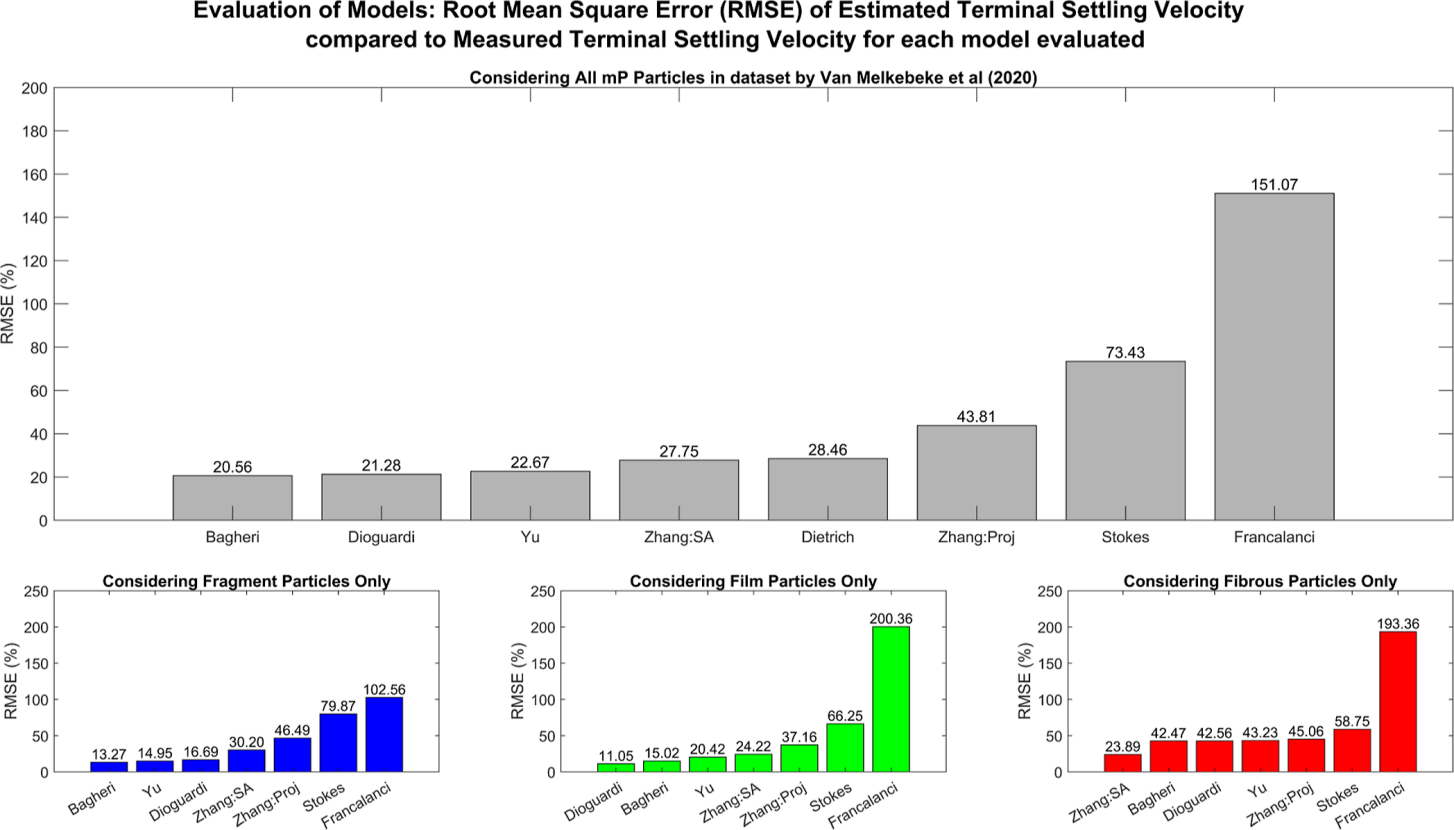
RMSE of the estimated terminal settling velocity for each model evaluated compared to the measured terminal settling velocity from the dataset by Van Melkebeke et al*.*^9^ The main figure illustrates the absolute average relative error for the entire dataset, while the lower figure shows the error when each morphology within the dataset is considered separately. The key for the models evaluated is Stokes = Stokes model^16^ using the particle surface area as the particle effective area, Bagheri = Bagheri and Bonadonna’s model^11^ using the particle projection area as the particle effective area, Dioguardi = Dioguardi et al.’s model^10^ using the particle projection area as the particle effective area, Zhang:SA = Zhang and Choi’s model^12^ using the particle surface area as the particle effective area, Zhang:Proj = Zhang and Choi’s model^12^ using the maximum cross-sectional area as the particle effective area, Dietrich = Dietrich’s model,^14^ Francalanci = Francalanci’s model,^13^ and Yu = Yu et al.’s model.^15^

All of the models evaluated were less accurate in reproducing the settling velocity of fibrous particles compared to film and fragment particles with the exception of Francalanci et al.^13^ and Stokes^16^ (Figures 2 and 3). Zhang and Choi’s^12^ model most closely predicts the terminal settling velocity of fibrous particles with an m value of 0.87 (Table 2) and |AE| of 19.14% (Table 4). This model was derived primarily to predict the terminal settling velocity of fibrous particles, and so it is expected that it is more suitable for this purpose. It also produces relatively consistent results for all morphologies considered, with m values of 0.86 and 0.88 (Table 2) and |AE|s of 26.11 and 20.40% (Table 4) for fragments and films, respectively.

Dioguardi et al.^10^ and Bagheri and Bonadonna’s^11^ models are noticeably less accurate in predicting the terminal settling velocity of fibers and produce an |AE| almost 3 times higher than the |AE| obtained when only fragments were considered at 36.45 and 34.59%, respectively (Table 4). This may have occurred since the dataset used during the derivation of these models did not contain particles that are analogous to fibrous particles. For example, the dataset used to derive Dioguardi et al.’s^10^ model contained natural particles with Dellino shape factors from 0.335 to 0.943, while the Dellino shape factor of the fibrous particles in the dataset used in this model evaluation ranged from 0.012 to 0.187. Yu et al.’s model^15^ which does take into account fibrous particles more accurately reproduces their measured terminal settling velocity, with an m value of 1.27 (Table 2) and an absolute average error of 33.18% (Table 4).

In addition, the approximated projected area of fibers used during the model evaluation is lower than their actual projected area since the most stable orientation of fibrous particles sinking in a fluid is the horizontal orientation with their maximum projection area normal to the direction of motion. The approximation used may therefore have contributed toward the reduced performance of the implicit models for fibrous particles.

Overall, the model evaluation shows that the drag models proposed by Dioguardi et al.,^10^ Bagheri and Bonadonna,^11^ and Yu et al.^15^ have a similarly high precision in predicting the terminal settling velocity of fragment and film particles but are less accurate for fibrous particles. As an explicit model, Yu et al.’s^15^ model is more computationally efficient than the implicit models that require an iterative method to calculate the terminal settling velocity and therefore may be most appropriate for implementation in an mP transport model. To further verify this, the performance of Yu et al.’s^15^ model was re-tested using an independent dataset.

### 3.2 Re-evaluation of Yu’s Model

**Figure 4:**
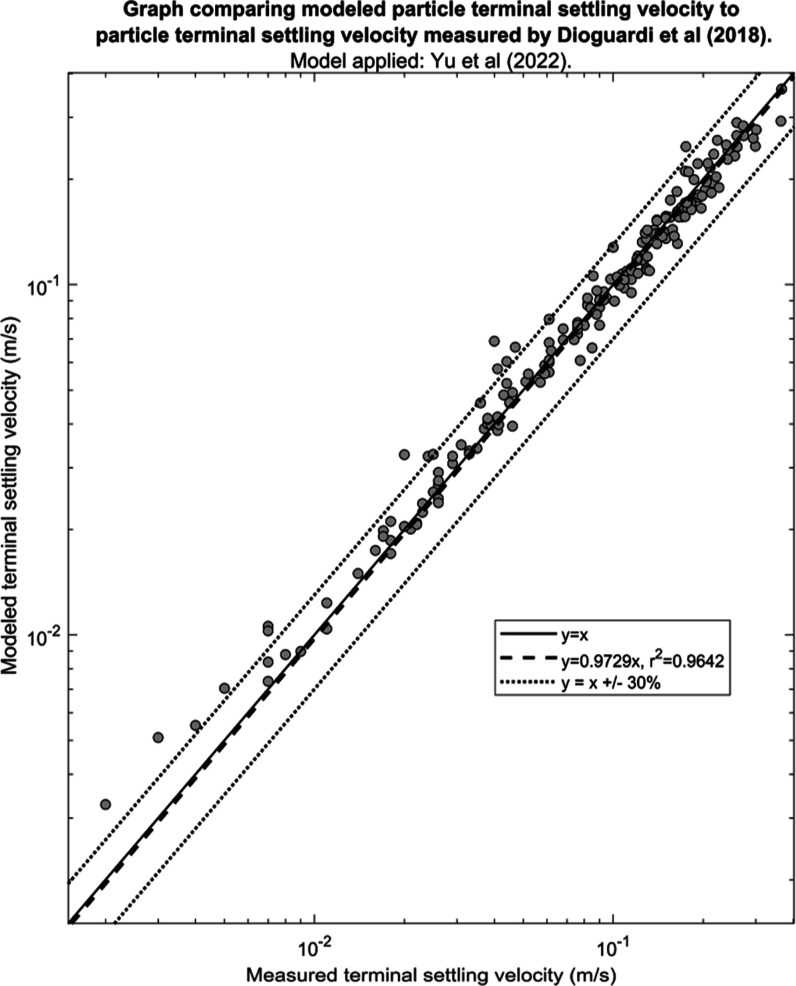
Output of the re-evaluation of the model by Yu et al.^15^ showing the model-estimated terminal settling velocity against the measured terminal settling velocity from the dataset in Dioguardi et al^10^ The solid line indicates the ideal fit where the modeled terminal settling velocity equals the measured terminal settling velocity, and the dotted lines indicate the modeled terminal settling velocity equals ±30% of the measured terminal settling velocity. The dashed line indicates the best fit line in the form *y* = *mx* that was obtained using linear regression.

The re-evaluation of Yu et al.’s model^15^ using the independent dataset from Dioguardi et al.^10^ demonstrates that the model closely estimates the terminal settling velocity of the particles, with *m* = 0.97 and *r*^2^ = 0.96 (Figure 4). The |AE| and the RMSE were also very low, at 3.11 and 16.03%, respectively (Table 5), indicating that the model is adequate at predicting the terminal settling velocity of irregularly shaped particles in a fluid.

**Table 5:** Results of Linear Regression and Error Analysis Undertaken during Re-evaluation of Yu et al.’s^15^ Model Using the Dataset from Dioguardi et al.^10^^,^ a

model	shape	|AE| (%)	RMSE (%)	*m*	*r*^2^
Yu et al. (2022)	All	10.27	16.03	0.97	0.96

a *m* is the gradient and *r*^2^ is the coefficient of determination of the fitted line of the form *y* = *mx* obtained using linear regression. |AE| is the average absolute relative error (%) and RMSE is the root-mean-square error (%).

### 3.3 Further Analysis

While Stokes law^16^ can be applied to calculate the terminal settling velocity of spherical particles, difficulties arise when estimating the terminal settling velocity of mP particles due to the wide range of morphologies they exhibit, including 3D shapes such as fragments, 2D shapes such as films, and 1D shapes such as fibers and lines. Obtaining an appropriate expression for the drag coefficient *C*_d_ and defining the effective area used to calculate the drag force of irregular particles are not straightforward tasks.

The effective area can either be taken as the particle surface area, which lends itself to understanding the drag force as friction acting on the body, or as the particle projection area, which would suggest that the drag force acts as resistance to the flow.^24^ The projection area is commonly used as the effective area for irregular particles as it is often more straightforward to measure than the surface area.^25^ The implicit models were tested using both the particle surface area and the projection area. The particle surface area was calculated by multiplying the particle sphericity, given in the dataset in Van Melkebeke et al.*,*^9^ by the surface area of the volume equivalent sphere. The projection area of the irregularly shaped particles was approximated using the projection area of the volume equivalent sphere as the dataset contained insufficient information for an accurate calculation.

The particle projection area is explicitly stipulated as the effective area during the derivation of Dioguardi et al.^10^ and Bagheri and Bonadonna’s^11^ models, and as a result, they produced less accurate results when implemented using the particle surface area. These results are not presented in this paper but are included in Supporting Information 9 for reference.

The accuracy of Stokes’^16^ model was reduced when the projection area was used as the effective area, with an overall absolute average error of 1171% compared to an error of 59.88% when the particle surface area was used. This may have occurred since the particle surface area is an order of magnitude higher than the approximated projection area, meaning that the drag force will be lower and the estimated terminal settling velocity will be higher when the projection area is used. These results are excluded from the analysis in this paper due to their low accuracy but are included in Supporting Information 9 for reference.

### 3.4 Impact of the Choice of Initial Velocity on the Result of the Implicit Models

When using an implicit model to calculate the terminal settling velocity, an initial value of the settling velocity must be specified to initiate the iterative calculation. To explore the impact of the choice of initial velocity on the modeled terminal settling velocity, the implicit models were run using six different initial velocity values ranging from 5 × 10^–6^ to 1 × 10^–3^ m/s. These values were chosen to ensure that the initial velocity was always less than the expected terminal settling velocity while also encompassing a wide range of values.

**Figure 5:**
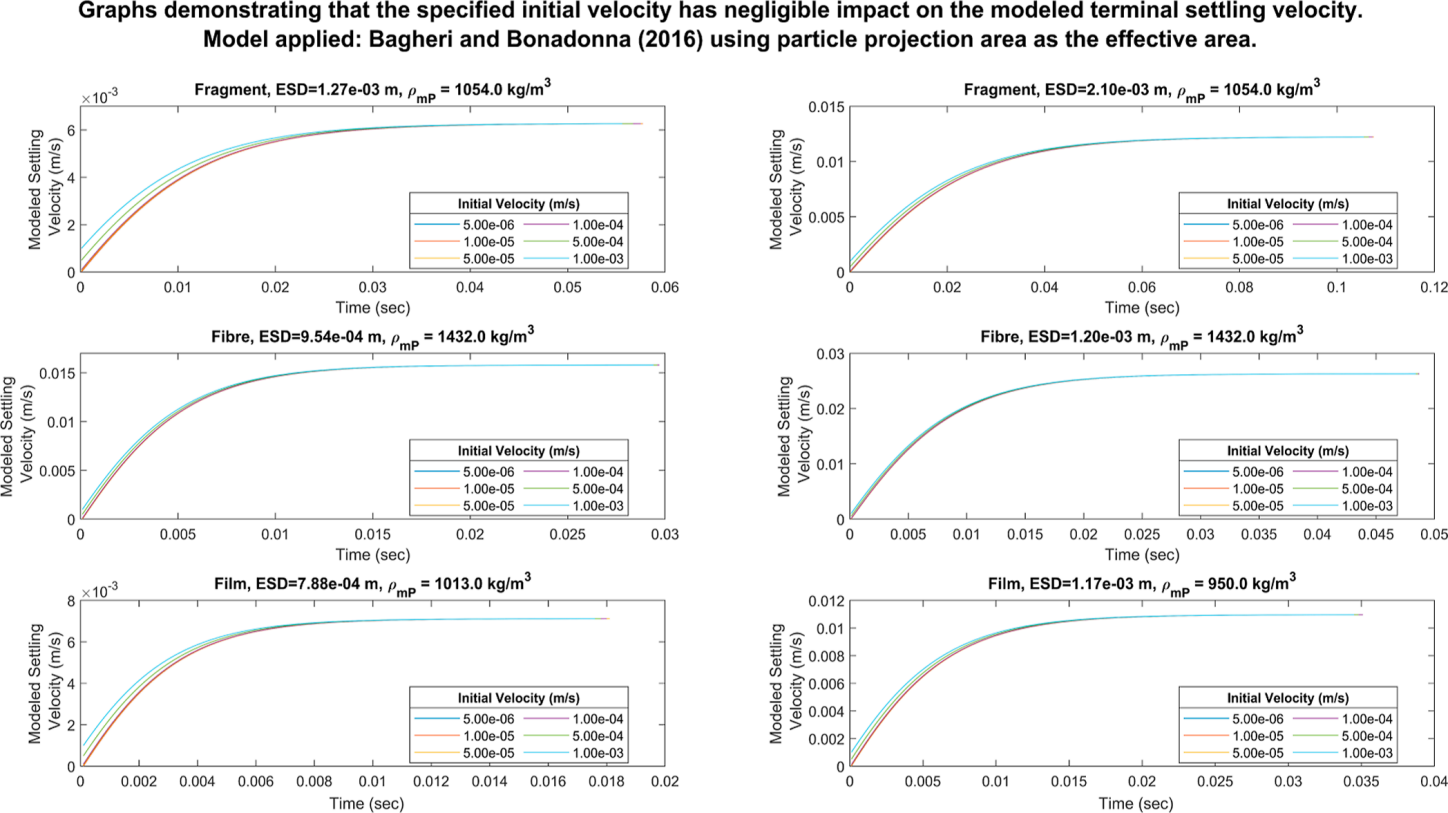
Impact of the choice of initial velocity on the modeled settling velocity when using Bagheri and Bonadonna’s model^11^ with the particle projection area as the effective area for six particles that were randomly extracted from the dataset by Van Melkebeke et al.^9^ The output from the remaining implicit models is included in Supporting Information 11.

The modeled settling velocity converged to the same terminal value during these tests, regardless of the initial velocity specified, indicating that the choice of initial velocity has no impact on the result of the implicit models. The main difference observed when varying the initial velocity was that the time taken to attain the terminal settling velocity decreased as the specified initial velocity approached the terminal settling velocity since less calculation iterations were required. This is illustrated in Figure 5 which presents the results from conducting this test on six randomly selected particles using the model by Bagheri and Bonadonna.^11^ The results for the other implicit models are included in Supporting Information 11.

Therefore, when implementing these models in a mP transport model, the choice of initial velocity will not be a critical factor provided that a realistic value is chosen.

### 3.5 Variation in the Terminal Settling Velocity over the Range of Density in the Ocean and the Impact on the Models of Assuming a Constant Terminal Settling Velocity

Seawater density is an input variable in all the models tested. Within the ocean, there is a vertical gradient of density, with lower density seawater at the surface and higher density seawater at depth in a stable water column. The density of 99% of seawater is within ±2% of the average value^26^ of 1030 kg/m^3^. To explore the influence of seawater density on the calculated terminal settling velocity, the models were tested using six fluid density values ranging from 1019 to 1050 kg/m^3^ to reflect the range of density encountered in the ocean.

The results of the test illustrate that the terminal settling velocity does not vary significantly over the expected range of seawater density for all models tested. However, when the ratio of seawater density to particle density approaches 1, the terminal settling velocity approaches zero. This occurs since there is zero net gravitational force acting on the mP when its density equals the seawater density and the mP is said to be neutrally buoyant.

**Figure 6:**
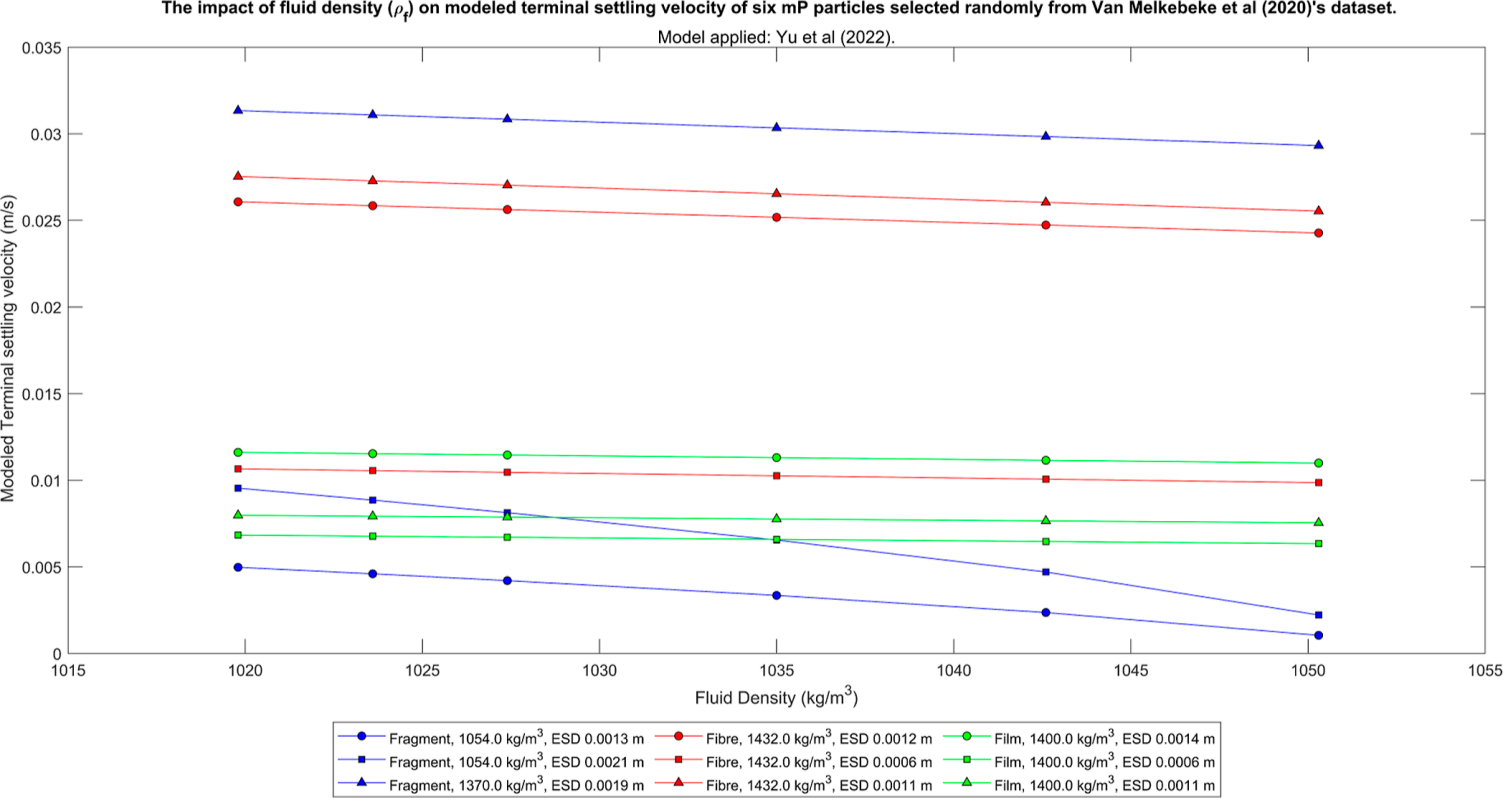
Output obtained when investigating the influence of fluid density on the terminal settling velocity of six random particles using the model by Yu et al*.*^15^ The output from the remaining implicit models is included in Supporting Information 12.

**Figure 7:**
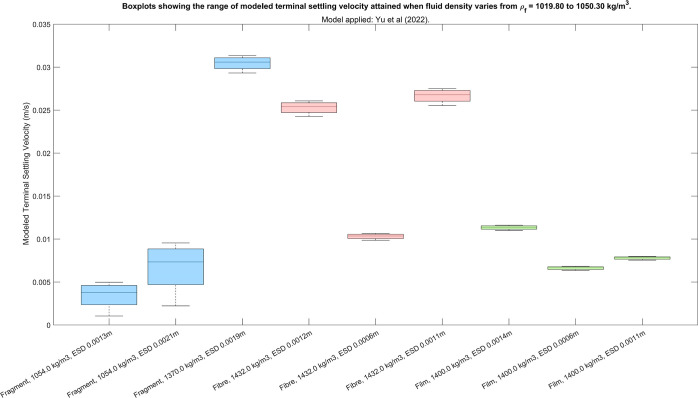
Range of settling velocity obtained for each of six random particles using the model by Yu et al.^15^ when the fluid density varied from 1019 to 1050 kg/m^3^. The output from the remaining implicit models is included in Supporting Information 12.

For example, when Yu et al.’s model^15^ was implemented at various seawater densities, the terminal settling velocity varied from 0.0004 to 0.002 m/s, excluding the particles which approached neutral buoyancy (Figures 6 and 7). This is equivalent to the particle traveling an additional 34–172 m per day at the lowest seawater density compared to the highest seawater density tested, which is a negligible distance compared to the overall depth of the ocean and spatial and temporal resolution of an ocean model.

Overall, the results of this test indicate that in a mP transport model, it is suitable to assume a constant terminal settling velocity regardless of the seawater density, provided that the model accounts for mPs attaining neutral buoyancy.

### 3.6 Exploring the Impact of Using a Constant Terminal Sinking Velocity on the Distance Traveled by the mPs

To further examine the impact of assuming a constant settling velocity over time on the results of an mP model, the distance traveled by the mP while attaining terminal settling velocity was compared to the distance traveled at the constant velocity during the same period. This exercise was only carried out for the implicit models as the explicit models do not provide information on particle behavior before the terminal settling velocity is attained.

**Figure 8:**
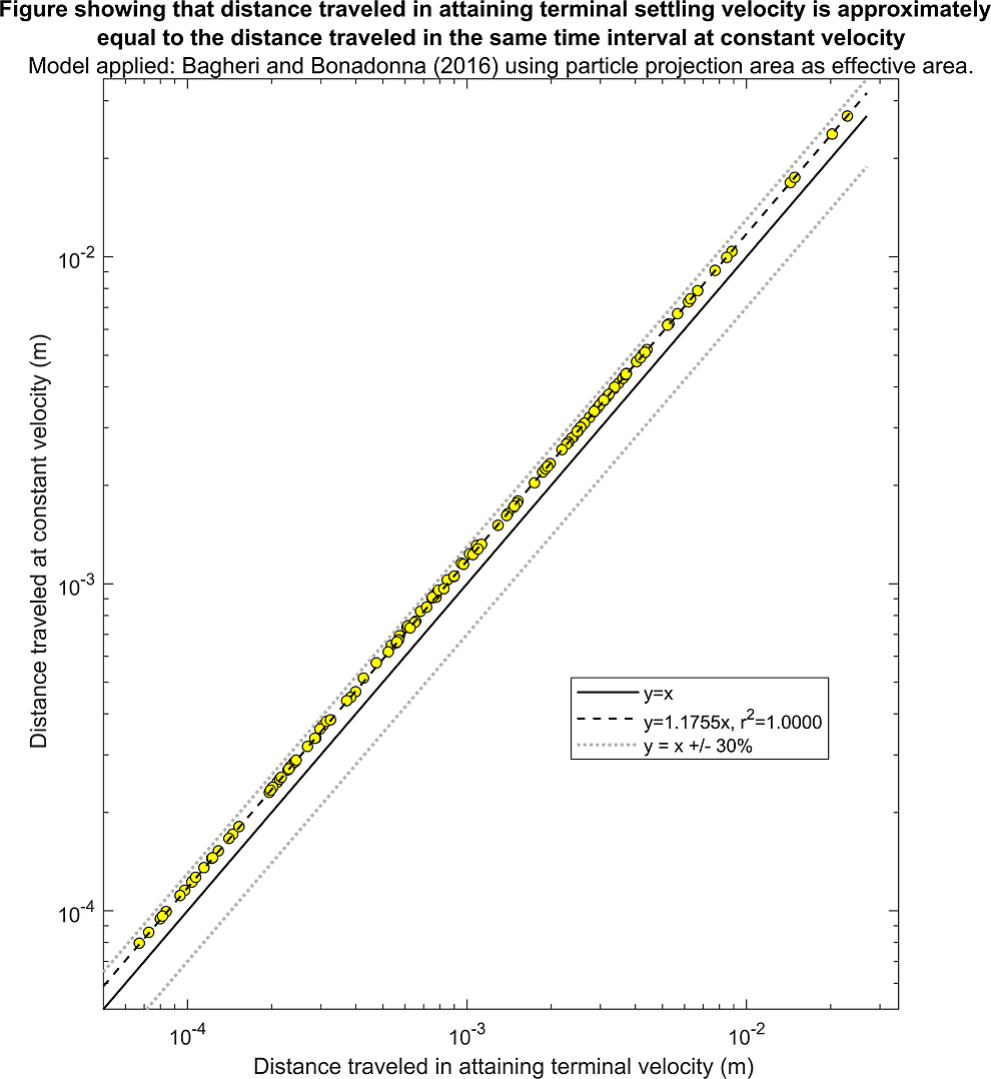
Comparison of the distance traveled in attaining the terminal settling velocity to the distance traveled if the particle sank constantly at the terminal settling velocity in the equivalent period of time when using the model by Bagheri and Bonadonna.^11^ The solid line indicates the ideal fit where there is no difference in the distance traveled, and the dotted lines indicate that the distance traveled at a constant velocity is ±30% of the distance traveled while attaining the terminal settling velocity. The dashed line indicates the best fit line in the form *y* = *mx* that was obtained using linear regression. The output from the remaining implicit models is included in Supporting Information 13 for reference.

As the particle accelerates until the terminal settling velocity is attained, the distance traveled at a constant velocity will always be slightly higher than the actual distance traveled. This is illustrated in the output for each of the models, which show that the distance traveled at a constant settling velocity is 14–18% higher than the actual distance traveled (Supporting Information 13). For example, when Bagheri and Bonadonna’s model^11^ was tested, the distance traveled at a constant velocity was 17.55% higher than the actual distance traveled (Figure 8). However, since the terminal settling velocity was attained in a relatively short space of time for all models considered, the distance traveled by the particles was very small. For example, the terminal settling velocity was attained after 0.07 sec on average when using Bagheri and Bonadonna’s model,^11^ and the distance traveled during this time ranged from 0.07 mm to 3 cm.

Overall, the time taken to attain terminal settling velocity and the distance traveled during this time are negligible compared to the timestep required in an mP transport model and the overall depth of the ocean. This suggests that in the context of models of mP transport in the marine environment which consider vertical transport due only to the sum of the gravitational, buoyant, and drag forces, it is suitable to assume that the particle settling velocity is constant over time and equal to the calculated terminal settling velocity. Therefore, in the absence of more accurate data on mP vertical transport in the marine environment, it may be appropriate to use the terminal settling velocity in realistic models of mP transport, provided that this is highlighted as a simplification and a model limitation.

### 3.7 Limitations to This Research

There are several limitations to this study. The models evaluated during the research were empirical, and it is not recommended that they are applied beyond the limits of the experimental data used in their derivation. However, during the evaluation, the models were at times tested beyond their limits, which may impact the accuracy of their results. This occurred primarily for the models that were derived for natural particles since they did not account for the wider range of mP morphologies such as films and fibers.

As noted in Section 2.3, an iterative method was used to calculate the terminal settling velocity in this research and evaluate the appropriateness of implicit settling velocity models in an mP modeling context where the terminal settling velocity of the particle is unknown. When Van Melkebeke et al.^9^ evaluated the models using the same dataset but by applying the non-iterative method which is outlined in Section 2.3, the evaluation produced different results. For example, Dioguardi et al.’s model^10^ produced better results during the non-iterative tests, with *m* = 0.99, |AE| = 13.20, and RMSE = 19.09. Dioguardi et al.^27^ also observed a very small change in the performance of the models evaluated, both positively and negatively, when they evaluated the performance of models using an iterative method compared to the non-iterative method. Therefore, while our study used the same dataset as in the study by Van Melkebeke et al.,^9^ it is likely that we would obtain different results when evaluating the same implicit models due to the difference in the method used to calculate the terminal settling velocity.

Finally, it is important to note that the evaluation in this paper considers only mP sinking in a quiescent water column due to the sum of the gravitational, buoyant, and drag forces. Consequently, the assumptions that were described to simplify the simulation of mP vertical transport in a modeling context are only valid in a model which does not consider any additional processes that impact the vertical transport of mPs, such as biofouling, weather events, and incorporation into biological aggregates.

## 4 Conclusions

Overall, the results indicate that the models by Bagheri and Bonadonna,^11^ Dioguardi et al.,^10^ and Yu et al.^15^ most accurately reproduce the measured terminal settling velocity of fragment and film particles. The model by Zhang and Choi,^12^ which was derived explicitly for fibrous particles, is most accurate in reproducing the terminal settling velocity of fibers. We recommend that Yu et al.’s explicit model^15^ is the most appropriate settling velocity model in the context of a mP transport model since it provided comparably accurate results to the best performing but more computationally expensive implicit models.

The additional tests identified that, when implementing the drag models in a mP transport model, the choice of initial velocity is not a critical factor, provided that a realistic initial velocity value is chosen. Furthermore, it is suitable to assume that the mP particles have a constant settling velocity over time since the terminal settling velocity is attained in a very short space of time. Finally, an evaluation of the influence of fluid density on the estimated terminal settling velocity demonstrated that the variation in mP terminal settling velocity across the expected seawater density range is negligible. Therefore, in an mP modeling context, it is suitable to assume a constant terminal settling velocity regardless of the seawater density, provided that the model will consider the impact of mPs attaining neutral buoyancy.

These findings improve our understanding on the implementation of drag models to determine the settling velocity of irregular particles in the context of mP transport models. However, additional processes which may be important in the vertical transport of mPs should also be considered in efforts to model mP transport, including wind-mixing,^17^ weather events,^18^ and the influence of biofouling.^19^ The settling velocity of mPs is a key parameter within mP transport models that influences their fate and bioavailability to organisms by controlling the residence time of mPs in the water column. Therefore, understanding the vertical transport of mPs is an important prerequisite to the completion of a comprehensive risk assessment of mPs in the ocean and the determination of their potential to cause significant ecological harm.

## Supporting Information Available

Additional model descriptions, experimental details, and results of tests on individual models as mentioned in the text (pdf)
