# Supplementary material for: Modeling Microplastic Transport in the Marine Environment: Testing Empirical Models of Particle Terminal Sinking Velocity for Irregularly Shaped Particles
Source: ACS ES T Water. 2023 Mar 22;3(4):984–95. doi: 10.1021/acsestwater.2c00466 (PMC10111405; doi:10.1021/acsestwater.2c00466)
Supplement: Supplementary file 1 — ew2c00466_si_001.pdf [file ew2c00466_si_001.pdf]

Supplementary information for:

Modelling microplastic (mP) transport in the marine environment: Testing empirical models of particle terminal sinking velocity for irregularly shaped particles.

Róisín Coyle, Matthew Service, Ursula Witte, Gary Hardiman, Jennifer McKinley

Contents:

SI 1. Description of empirical models evaluated. 2

SI 2: Procedure to implement the explicit model by Stokes (1851)[1] 5

SI 3: Procedure to implement the explicit model by Bagheri and Bonadonna (2016)[4] 7

SI 4: Procedure to implement the explicit model by Dioguardi *et al.* (2018)[5] 9

SI 5: Procedure to implement the explicit model by Zhang and Choi (2021)[7] 11

SI 6: Procedure to implement the implicit model by Dietrich (1982)[9] 13

SI 7: Procedure to implement the implicit model by Francalanci *et al.* (2021)[10] 15

SI 8: Procedure to implement the implicit model by Yu *et al.* (2022)[11] 17

SI 9: Results of all morphologies for each implicit model tested during the evaluation of the empirical drag models. 18

SI 10: Results of all morphologies for each explicit model tested during the evaluation of the empirical drag models. 26

SI 11: Results for each model tested during the evaluation of the impact of the choice of initial velocity on the result of the implicit models. 31

SI 12: Results for each model tested during the evaluation of the variation in the terminal settling velocity over the range of density in the ocean and the impact on the models of assuming a constant terminal settling velocity. 34

S1 13: Results for each model tested during the evaluation of the impact of using a constant terminal sinking velocity on the distance travelled by the mPs. 41

Works cited: 44

SI 1. Description of empirical models evaluated.

The existing empirical models for particle terminal sinking velocity that were evaluated during this research are presented and discussed in relation to the limitations and method applicability to modelling the fate and impacts of mPs within the marine environment. The empirical models are classed as either implicit or explicit depending on their mode of calculating the terminal settling velocity.

Implicit Models

The empirical models that give an expression for the drag coefficient C_D are classed as implicit models. In this study, an iterative method is then applied to calculate the terminal settling velocity in which the drag coefficient C_D is used to calculate the drag force and the particle acceleration is then calculated using the net vertical force. The process is repeated until the acceleration becomes negligible, at which point it is assumed the terminal settling velocity has been attained.

Stokes' law[1]states that the viscous force acting on a spherical body falling through a fluid depends directly on the particle radius, r, and velocity, w, and the viscosity of the fluid, μ. Using dimensional analysis and empirical results, Stokes proved that for spherical particles the drag force F_D can be expressed as $F_D = 6\pi r w \mu$ and used this knowledge to define the following expression for the drag coefficient: $C_D = \frac{24}{Re}$, where Re is the particle Reynolds number (further information on the derivation is included in SI 2). Stokes' law is used as the reference law for spheres during this evaluation but is expected to overestimate the terminal settling velocity of non-spherical particles which will experience a higher drag force than the equivalent spherical particles. The expression is also only valid in the Stokes Regime where Re<0.1 and starts to fail when Re>1 [2, 3]. Therefore, Stokes law may be used to estimate the terminal settling velocity of spherical mP particles but an alternative model may be more appropriate for irregular particles.

Bagheri and Bonadonna[4] developed an empirical model to predict the drag coefficient of non-spherical irregularly and regularly shaped solid particles falling in a fluid based on the particle Reynolds number, shape and orientation and the particle-to-fluid density ratio. The expression proposed for the drag coefficient is as follows:

$$C_D = \frac{24k_s}{Re}\left[1 + 0.125\left(\frac{Re\,k_N}{k_s}\right)^{\frac{2}{3}}\right] + \frac{0.46k_N}{1+\frac{5330}{\left(\frac{Re\,k_N}{k_s}\right)}}$$

Where Re is the particle Reynolds number, K_S is the Stokes drag correction and K_N is the Newtons drag correction. The drag corrections were parameterised experimentally, and the steps required for their calculation are outlined in SI 3. As this model and all the correlations within it are derived empirically, it is not recommended that it is applied beyond the limits of the experimental data. The data was collected using both a settling column and a wind tunnel and so the model is valid in a range of fluid regimes (Re<<1 to Re<$3\text{x}10^5$). The particles used during the settling column experiments had a large size range, from 155μm to 1.8mm in the settling column experiments and 10.9mm to 61.2mm in the wind tunnel experiments, and covered a range of morphologies, including irregular volcanic particles, cylinders, ellipsoids, discs, and parallelepipeds.

The implicit model by Dioguardi *et al.*[5] presents an empirical expression for the drag coefficient that is dependent on the particle shape. The resulting expression for the drag coefficient is: $C_{d,calc} = \frac{24}{Re}\left(\frac{1-\Psi}{Re}+1\right)^{0.25} + \frac{24}{Re}(0.1806Re^{0.6459})\Psi^{-(Re^{0.08})} + \frac{0.4251}{1+\frac{6880.95}{Re}\Psi^{5.05}}$

Where Re is the particle Reynolds number and Ψ is the Dellino Shape Factor, which is the ratio of the particle sphericity to the particle circularity. The steps required for the calculation of these parameters are outlined in SI 4. The expression converges to the drag coefficient of a perfect sphere given by Haider and Levenspiel[6] when Ψ=1. As with the model by Bagheri and Bonadonna[4], the model by Dioguardi *et al.*[5] should not be applied beyond the tested conditions. As the database used to derive the model includes Reynolds numbers ranging from 0.03 to 1x10^5 and particles with Dellino shape factors from 0.335 to 0.943, it is valid over a range of fluid regimes and applicable to irregular particle shapes.

The newest implicit model considered was developed by Zhang and Choi[7] and proposes that the Aschenbrenner shape factor is used to calculate the drag coefficient to more readily distinguish the behaviour of fibrous particles. Their expression for the drag coefficient was derived using linear regression and is as follows: $C_D = \frac{58.58AS^{\ 0.1936}}{Re^{0.8273}}$ where Re is the particle Reynolds number and ASF is the Aschenbrenner shape factor. The process required to implement this model to calculate the terminal settling velocity, including the steps required to estimate the required particle properties, is outlined in SI 5. This model was derived using 70% of the fibre data in the dataset by Van Melkebeke *et al.*[8], for which the Reynolds number varies from 6.9 to 3630. It has been shown that, as well as fibres, the model reasonably predicts the settling velocity of fragment and film particles, but it is not suited to predicting the settling velocity of weathered particles.

Explicit Models

Empirical models which give an expression to directly calculate the terminal settling velocity of particles falling in a fluid are known as explicit models. These models are computationally more efficient than implicit models since they do not require an iterative calculation.

Dietrich[9] developed an empirical equation to explicitly calculate the settling velocity of natural particles using the results of settling velocity experiments. The model expresses separately and quantitatively the effects of the particle density, size, shape, and roundness on the particle settling velocity but has no dependence on the particle Reynolds number Re. The final equation expresses the settling velocity in its dimensionless form and is stated to be reasonably accurate in calculating the settling velocity of natural particles:

$$W_* = R_3 10^{R_1+R_2}$$

Where W_* is the dimensionless settling velocity, R_1 is a fitted equation for particle size, R_2 is a fitted equation for particle shape and R_3 is a fitted equation for particle roundness. The definition of these equations for the implementation of the model is outlined in the SI 6. The model uses the Corey Shape Factor (CSF) to quantify particle shape. However, due to the definition of the fitted equation for particle shape R_2, the model fails when CSF<0.15 and therefore is not recommended for use for particles with CSF<0.2.

A method to explicitly predict the settling velocity of plastic and natural particles of different shapes, ranging from one dimensional (fibres) to three dimensional (pellets, spheres), across a range of flow regimes was developed empirically by Francalanci *et al.*[10] and validated using an independent dataset. The terminal settling velocity is expressed in its dimensionless form as:

$$W_* = \frac{{D_*}^2}{C_1 + \left(0.75 C_2 {D_*}^3\right)^n}$$

Where W_* is the dimensionless settling velocity, D_* is the dimensionless particle size, C_1 and C_2 are coefficients, and n is an exponent which was obtained during calibration of the equation. The full procedure required to implement this model is outlined in SI 7. The experiment included particles with 1D, 2D and 3D morphologies with sizes of 1.68 to 5.44mm and so the model is applicable to a wide range of particle shapes and sizes. However, the model is limited in that it only considers quiescent fluids and neglects the effect of a bulk of particles on the settling velocity. The model is independent of the particle Reynolds number.

An alternative drag law was developed by Yu *et al.*[11] to explicitly calculate the settling velocity using the Corey Shape Factor (CSF) and the particle sphericity Φ. This method provides an expression for the drag coefficient C_d which can be used to calculate the terminal settling velocity with the following equation:

$$w_s = \left(\nu g \frac{\rho_s - \rho_f}{\rho_f}\right)^{\frac{1}{3}} \sqrt{\frac{4 d_*}{3 C_d}}$$

Where w_s is the particle settling velocity, ν is the fluid viscosity, g is the gravitational acceleration, ρ_s is the particle density, ρ_f is the fluid density, d_* is the dimensionless particle diameter and C_d is the drag coefficient proposed by Yu *et al.*[11]. The process to implement this model, including the calculation of the required parameters, is outlined in SI 8. As the model was derived using data that lies predominantly in the transitional regime (1<Re<1000), its applicability to the Laminar regime (Re<1) and turbulent regime (Re>1000) is uncertain. Since the dataset used to derive the model contained 699 mPs ranging in size from 0.0025mm to 0.0356mm and included pellets, cylinders, spheres, fragments, fibres, fishing lines and angular and nodular particles, the model is applicable to a wide range of particle properties.

SI 2: Procedure to implement the explicit model by Stokes (1851)[1]

Derivation:

Stokes' law states that the viscous force acting on a spherical body falling through a fluid depends directly on the radius r (m), and velocity w (m/s) of the sphere and the viscosity μ ($kgm^{-1}s^{-1}$) of the liquid:

$$F \propto r^a w^b \mu^c$$

Using dimensional analysis, it can be shown that:

$$F = krw\mu$$

Where k is a coefficient of proportionality, which has been shown empirically to equal 6π. Therefore, the viscous drag force is:

$$F = 6\pi rw\mu$$

Equating this to the expression for drag force containing the non-dimensional drag coefficient C_D gives:

$$6\pi rw\mu = \frac{1}{2}\rho_w S w^2 C_D$$

Where S is the effective area of the particle (m^2), ρ_w is the fluid density (kg/m^3) and w is the particle settling velocity (m/s). For a spherical particle, defining the effective area as the projected area[3] gives $S = \pi r^2$:

$$6\pi rw\mu = \frac{1}{2}\rho_w \pi r^2 w^2 C_D$$

Which results in the expression:

$$C_D = \frac{24}{Re}$$

Where Re is the dimensionless Reynold's number $Re = \frac{\rho_w wd}{\mu}$.

Implementation:

1. Read in microplastic (mP) dataset from Van Melkebeke *et al*[8].
2. Calculate additional required particle properties:
    a. Surface area of equivalent sphere (m^2) $SA_{EqS} \quad = 4\pi\left(\frac{d_{equi}}{2}\right)^2$
    b. mP surface area (m^2) $SA_{mP} = \frac{SA_{EqSPh}}{\Phi}$
    c. Equivalent spherical volume (m^3) $Vol_{mP} = \frac{4}{3}\pi\left(\frac{d_{equi}}{2}\right)^3$
    d. mP mass (kg) $Mass_{mP} = \rho_{mP} Vol_{mP}$
    e. Projected area of volume equivalent sphere (m^2) $ProjA_{ESD} = \pi\left(\frac{d_{equi}}{2}\right)^2$
3. Specify the timestep (sec).
4. Specify the initial velocity at time t=0 in m/s.
5. For each particle at each time step:
    a. Calculate the Reynolds number (dimensionless) $Re = \frac{\rho_w wd}{\mu}$
    b. Calculate the drag coefficient (dimensionless) using Stokes model: $C_D = \frac{24}{Re}$

c. Calculate the drag force (N) $F_d = \frac{1}{2}\rho_w S w^2 Cd$
d. Calculate the gravitational force (N) $F_g = Vol_{mP}\rho_{mP}g$
e. Calculate the buoyant force (N) $F_b = Vol_{mP}\rho_W g$
f. Calculate the net force acting on the mP (N): $F_{net} = F_g - F_b - F_d$
g. Calculate the settling velocity at the next time step (m/s):

$$w^{t+1} = \left(\frac{F_{net}}{Mass_{mP}}\Delta t\right) + w^t$$

h. Calculate the acceleration (m/s^2):

$$Acceleration = \frac{w^{t+1} - w^t}{\Delta t}$$

   i. If acceleration>0.001m/s^2, step forward one timestep and restart the loop at point (5a).
   ii. If acceleration<0.001m/s^2, assume that terminal settling velocity has been obtained and end the calculation iterations.

SI 3: Procedure to implement the explicit model by Bagheri and Bonadonna (2016)[4]

Description

The expression for the drag coefficient of non-spherical irregularly and regularly shaped solid particles falling in a fluid (gas or liquid) is:

$$C_D = \frac{24k_s}{Re}\left[1 + 0.125\left(\frac{Re\,k_N}{k_s}\right)^{\frac{2}{3}}\right] + \frac{0.46k_N}{1 + \frac{5330}{\left(\frac{Re\,k_N}{k_s}\right)}}$$

Where Re is the particle Reynolds number $Re = \frac{\rho_f w d_{equi}}{\mu}$.

The shape descriptors particle flatness, f, and elongation, e, are used in the model to quantify particle shape since they are straightforward to measure and calculate:

$$f = \frac{S}{I}$$

$$e = \frac{I}{L}$$

Where S, I and L are the shortest, intermediate, and longest length of the particle. These descriptors are then used to define the two new shape descriptors: Stokes form factor F_S and Newtons form factor F_N:

$$F_S = f e^{1.3}\left(\frac{{d_{eq}}^3}{LIS}\right)$$

$$F_N = f^2 e\left(\frac{{d_{eq}}^3}{LIS}\right)$$

The experimental results demonstrated that the shape dependent Stokes drag correction k_s correlates with the Stokes form factor F_S as below:

$$k_s = \frac{1}{2}\left({F_S}^{\frac{1}{3}} + {F_S}^{-\frac{1}{3}}\right)$$

Non-linear regression was used to derive the correlation of the shape dependent Newtons drag correction k_N with Newtons form factor F_N and the particle to fluid density ratio ρ' $\left(\rho' = \frac{\rho_p}{\rho_f}\right)$:

$$k_N = 10^{\alpha_2[-\log F_N]^{\beta_2}}$$

Where α_2 and β_2 are functions of ρ':

$$\alpha_2 = 0.45 + \left[\frac{10}{(\exp(2.5\log\rho') + 30)}\right]$$

$$\beta_2 = 1 - \left[\frac{37}{(\exp(3\log\rho') + 100)}\right]$$

Implementation:

1. Read in mP dataset from Van Melkebeke *et al*[8].
2. Calculate additional required particle properties:
    a. Surface area of equivalent sphere (m^2): $SA_{EqSph} = 4\pi\left(\frac{d_{equi}}{2}\right)^2$
    b. mP surface area (m^2): $SA_{mP} = \frac{SA_{EqSPh}}{\Phi}$
    c. Equivalent spherical volume (m^3): $Vol_{mP} = \frac{4}{3}\pi\left(\frac{d_{equi}}{2}\right)^3$
    d. mP mass (kg): $Mass_{mP} = \rho_{mP} Vol_{mP}$
    e. Relative density (dimensionless): $\rho_{rel} = \frac{\rho_{mP} - \rho_w}{\rho_w}$
    f. Projected area of volume equivalent sphere (m^2): $ProjA_{ESD} = \pi\left(\frac{d_{equi}}{2}\right)^2$
3. Specify the timestep (sec).
4. Specify the initial velocity at time t=0 in m/s.
5. For each particle:
    a. Calculate the Stokes form factor (dimensionless): $F_S = fe^{1.3}\left(\frac{d_{eq}{}^3}{LIS}\right)$
    b. Calculate the Newtons form factor (dimensionless): $F_N = f^2 e\left(\frac{d_{eq}{}^3}{LIS}\right)$
    c. Calculate the shape dependent Stokes correction (dimensionless):
    $$k_s = \frac{1}{2}\left(F_S{}^{\frac{1}{3}} + F_S{}^{-\frac{1}{3}}\right)$$
    d. Calculate the density ratio (dimensionless): $\rho' = \frac{\rho_p}{\rho_f}$
    e. Calculate $\alpha_2 = 0.45 + \left[\frac{10}{(\exp(2.5\log\rho') + 3\ \ )}\right]$
    f. Calculate $\beta_2 = 1 - \left[\frac{37}{(\exp(3\log\rho') + 100)}\right]$
    g. Calculate the shape dependent Newton's drag correction (dimensionless) $k_N = 10^{\alpha_2[-\log F_N]^{\beta_2}}$
    h. For each time step:
        i. Calculate the Reynolds number (dimensionless) $Re = \frac{\rho v d}{\mu}$
        ii. Calculate the drag coefficient (dimensionless) using Bagheri's model:
        $$C_D = \frac{24k_s}{Re}\left[1 + 0.125\left(\frac{Re\ k_N}{k_s}\right)^{\frac{2}{3}}\right] + \frac{0.46k_N}{1 + \frac{5330}{\left(\frac{Re\ k_N}{k_s}\right)}}$$
        iii. Calculate the drag force (N): $F_d = \frac{1}{2}\rho_w S w^2 C_d$
        iv. Calculate the gravitational force (N): $F_g = Vol_{mP}\rho_{mP} g$
        v. Calculate the buoyant force (N): $F_b = Vol_{mP}\rho_W g$
        vi. Calculate the net force acting on the mP (N): $F_{net} = F_g - F_b - F_d$
        vii. Calculate the settling velocity at the next time step (m/s):
        $$w^{t+1} = \left(\frac{F_{net}}{Mass_{mP}}\Delta t\right) + w^t$$
        viii. Calculate the acceleration (m/s^2): $Acceleration = \frac{w^{t+1} - w^t}{\Delta t}$
            1. If acceleration>0.001m/s^2, step forward one timestep and restart the loop at point (5a).
            2. If acceleration<0.001m/s^2, assume that terminal settling velocity has been obtained and end the calculation iterations.

SI 4: Procedure to implement the explicit model by Dioguardi *et al.* (2018)[5]

Description

The expression proposed for the drag coefficient is:

$$C_{d,calc} = \frac{24}{Re}\left(\frac{1-\Psi}{Re}+1\right)^{0.25} + \frac{24}{Re}(0.1806Re^{0.6459})\Psi^{-(Re^{0.08})} + \frac{0.4251}{1+\frac{6880.95}{Re}\Psi^{5.05}}$$

Where Re is the particle Reynolds number $Re = \frac{\rho_f w d_{equi}}{\mu}$ and Ψ is the Dellino[12] shape factor which is expressed as:

$$\Psi = \frac{\Phi}{\mathrm{X}}$$

$$\Phi = \frac{A_{sph}}{A_p}$$

$$\mathrm{X} = \frac{P_{mp}}{P_c}$$

where Φ is the particle sphericity (dimensionless), X is the particle circularity (dimensionless), A_{sph} is the surface area of the volume equivalent sphere (m^2), A_p is the particle surface area (m^2), P_{mp} is the perimeter of the maximum projection area (m) and P_c is the perimeter of the circle equivalent to the maximum projection area A_{mp} of the particle (m).

Implementation

1. Read in mP dataset from Van Melkebeke *et al*[8].
2. Calculate additional required particle properties:
    a. Surface area of equivalent sphere (m^2): $SA_{EqSph} = 4\pi\left(\frac{d_{equi}}{2}\right)^2$
    b. mP surface area (m^2): $SA_{mP} = \frac{SA_{EqSPh}}{\Phi}$
    c. Equivalent spherical volume (m^3): $Vol_{mP} = \frac{4}{3}\pi\left(\frac{d_{equi}}{2}\right)^3$
    d. mP mass (kg): $Mass_{mP} = \rho_{mP}Vol_{mP}$
    e. Corey Shape factor (dimensionless): $CSF = \frac{c}{\sqrt{ab}}$
    f. Projected area of volume equivalent sphere (m^2): $ProjA_{ESD} = \pi\left(\frac{d_{equi}}{2}\right)^2$
3. Specify the timestep (sec).
4. Specify the initial velocity at time t=0 in m/s.
5. For each particle at each time step:
    a. Calculate the Reynolds number (dimensionless) $Re = \frac{\rho v d}{\mu}$
    b. Calculate the drag coefficient (dimensionless) using Dioguardi's model: $C_{d,calc} = \frac{24}{Re}\left(\frac{1-\Psi}{Re}+1\right)^{0.25} + \frac{24}{Re}(0.1806Re^{0.6459})\Psi^{-(Re^{0.08})} + \frac{0.4251}{1+\frac{6880.95}{Re}\Psi^{5.05}}$
    c. Calculate the drag force (N): $F_d = \frac{1}{2}\rho_w S w^2 C_{d,calc}$
    d. Calculate the gravitational force (N): $F_g = Vol_{mP}\rho_{mP}g$
    e. Calculate the buoyant force (N): $F_b = Vol_{mP}\rho_W g$
    f. Calculate the net force acting on the mP (N): $F_{net} = F_g - F_b - F_d$

g. Calculate the settling velocity at the next time step (m/s):

$$w^{t+1} = \left(\frac{F_{net}}{Mass_{mP}}\Delta t\right) + w^t$$

h. Calculate the acceleration (m/s^2):

$$Acceleration = \frac{w^{t+1} - w^t}{\Delta t}$$

   i. If acceleration>0.001m/s^2, step forward one timestep and restart the loop at point (5a).
   ii. If acceleration<0.001m/s^2, assume that terminal settling velocity has been obtained and end the calculation iterations.

SI 5: Procedure to implement the explicit model by Zhang and Choi (2021)[7]

Description

The proposed drag model is as follows:

$$C_D = \frac{58.58ASF^{0.1936}}{Re^{0.8273}}$$

Where Re is the particle Reynolds number $Re = \frac{\rho_f w d_{equi}}{\mu}$ and ASF is the Aschenbrenner shape factor $ASF = \frac{ac}{b^2}$, a is the longest length of the particle (m), b is the intermediate length of the particle (m) and c is the shortest length of the particle (m).

Implementation

1. Read in mP dataset from Van Melkebeke *et al*[8].
2. Calculate additional required particle properties:
   a. Surface area of equivalent sphere (m^2): $SA_{EqSph} = 4\pi\left(\frac{d_{equi}}{2}\right)^2$
   b. mP surface area (m^2): $SA_{mP} = \frac{SA_{EqSP}}{\Phi}$
   c. Equivalent spherical volume (m^3): $Vol_{mP} = \frac{4}{3}\pi\left(\frac{d_{equi}}{2}\right)^3$
   d. mP mass (kg): $Mass_{mP} = \rho_{mP}Vol_{mP}$
   e. Projected area of volume equivalent sphere (m^2): $ProjA_{ESD} = \pi\left(\frac{d_{equi}}{2}\right)^2$
   f. Zhang's estimated volume (m^3): $Vol_{ZC,Est} = abc$
   g. Zhang's volume equivalent spherical diameter (m): $D_{equiZC} = \sqrt[3]{\frac{6Vol_{ZC,Est}}{\pi}}$
   h. Zhang's equivalent diameter based on $ProjA_{ESD}$ (m): $d_{equi,ZC} = \sqrt{\frac{4a}{\pi}}$
   i. Zhang's projected area (m^2): $ProjA_{ZC} = \pi\left(\frac{d_{equi,ZC}}{2}\right)^2 = ab$
   j. Zhang's estimated mass (kg): $Mass_{mP} = Vol_{ZC,Est}\rho_{mP}$
3. Specify the timestep (sec).
4. Specify the initial velocity at time t=0 in m/s.
5. For each particle:
   a. Calculate the Aschenbrenner shape factor (dimensionless): $ASF = \frac{ac}{b^2}$.
   b. For each time step:
      i. Calculate the Reynolds number (dimensionless): $Re = \frac{\rho v d}{\mu}$
      ii. Calculate the drag coefficient using Zhang's model (dimensionless): $C_d = \frac{58.58ASF^{0.1936}}{Re^{0.8273}}$
      iii. Calculate the drag force (N): $F_d = \frac{1}{2}\rho_w S w^2 C_d$
      iv. Calculate the gravitational force (N): $F_g = Vol_{mP}\rho_{mP}g$
      v. Calculate the buoyant force (N): $F_b = Vol_{mP}\rho_W g$
      vi. Calculate the net force acting on the mP (N): $F_{net} = F_g - F_b - F_d$
      vii. Calculate the settling velocity at the next time step (m/s):

$$w^{t+1} = \left(\frac{F_{net}}{Mass_{mP}}\Delta t\right) + w^t$$

viii. Calculate the acceleration (m/s^2):

$$Acceleration = \frac{w^{t+1} - w^{t}}{\Delta t}$$

1. If acceleration>0.001m/s^2, step forward one timestep and restart the loop at point (5a).
2. If acceleration<0.001m/s^2, assume that terminal settling velocity has been obtained and end the calculation iterations.

SI 6: Procedure to implement the implicit model by Dietrich (1982)[9]

Derivation:

Dietrich[9] expresses the dimensionless settling velocity W_* as:

$$W_* = R_3 10^{R_1+R_2}$$

Where R_1 is a fitted equation for particle size:

$$R_1 = -3.76715 + 1.92944(\log D_*) - 0.09815(\log D_*)^{2.0} - 0.00575(\log D_*)^{3.0} + 0.00056(\log D_*)^{4.0}$$

R_2 is a fitted equation for particle shape:

$$R_2 = \left(\log\left(1 - \frac{1-CSF}{0.85}\right)\right) - (1 - CSF)^{2.3}\tanh(\log D_* - 4.6) + 0.3(0.5 - CSF)(1 - CSF)^{2.0}(\log D_* - 4.6)$$

R_3 is a fitted equation for particle roundness:

$$R_3 = \left[0.65 - \left(\frac{CSF}{2.83}\tanh(\log D_* - 4.6)\right)\right]^{\left(1+\frac{3.5-P}{2.5}\right)}$$

The nondimensional parameters used in these expressions are:

Corey Shape Factor CSF:

$$CSF = \frac{c}{\sqrt{ab}}$$

Where a, b and c are the longest, intermediate and shortest axis of the particle. Note that a, b and c are perpendicular. The CSF varies from 0.0 to 1.0, with a smaller CSF indicating a flatter particle.

Powers roundness index, P, which is determined by observation and varies from 0.0 (perfectly angular) to 6.0 (perfectly round).

Dimensionless particle size D_*:

$$D_* = \frac{(\rho_s - \rho)gD_n{}^3}{\rho v^2}$$

Where ρ_s is the particle density (kg/m^3), ρ is the fluid density (kg/m^3), D_n is the nominal spherical diameter i.e., the diameter of a sphere with the same volume as the particle (m) and v is the kinematic viscosity of the fluid (m^2/s).

Using the dimensionless settling velocity W_*, the particle settling velocity w_s (m/s) can then be calculated as follows:

$$W_* = \frac{\rho w_s^3}{(\rho_s - \rho)gv}$$

Implementation

1. Read in the mP dataset from Van Melkebeke *et al*[8].
2. Calculate additional required particle properties:
   a. Equivalent spherical volume (m^3): $Vol_{mP} = \frac{4}{3}\pi\left(\frac{d_{equi}}{2}\right)^3$
   b. mP mass (kg): $Mass_{mP} = \rho_{mP}Vol_{mP}$
   c. Corey Shape factor (dimensionless): $CSF = \frac{c}{\sqrt{ab}}$
   d. Relative density (dimensinless) $\rho_{rel} = \frac{\rho_{mP}-\rho_w}{\rho_w}$
3. For each particle:
   a. Calculate the dimensionless particle size $D_* = \frac{(\rho_s-\rho)gD_{equi}{}^3}{\rho v^2}$
   b. Calculate the fitted equation for particle size:
   $$R_1 = -3.76715 + 1.92944(\log D_*) - 0.09815(\log D_*)^{2.0} - 0.00575(\log D_*)^{3.0} + 0.00056(\log D_*)^{4.0}$$
   c. Calculate the fitted equation for particle shape:
   $$R_2 = \left(\log\left(1 - \frac{1-CSF}{0.85}\right)\right) - (1-CSF)^{2.3}\tanh(\log D_* - 4.6) + 0.3(0.5 - CSF)(1-CSF)^{2.0}(\log D_* - 4.6)$$
   d. Calculate the fitted equation for particle roundness:
   $$R_3 = \left[0.65 - \left(\frac{CSF}{2.83}\tanh(\log D_* - 4.6)\right)\right]^{\left(1+\frac{3.5-P}{2.5}\right)}$$
   e. Calculate the dimensionless particle velocity: $W_* = R_3 10^{R_1+R_2}$
   f. Calculate the actual settling velocity (m/s): $w_s^3 = \frac{W_*(\rho_s-\rho)gv}{\rho}$

SI 7: Procedure to implement the implicit model by Francalanci *et al.* (2021)[10]

Description

Francalanci *et al.*,[10] developed the following expression to calculate the dimensionless settling velocity of irregular particles:

$$W_* = \frac{{D_*}^2}{C_1 + \left(0.75 C_2 {D_*}^3\right)^n}$$

Where C_1 and C_2 are coefficients, E is a shape factor and n is an exponent which is obtained by calibration of the equation. These are calculated as follows:

$$C_1 = 18\, E^{-0.38}$$

$$E = a\left(\frac{a^2 + b^2 + c^2}{3}\right)^{-\frac{1}{2}}$$

$$C_2 = 0.3708\, CSF^{-0.1602}$$

$$n = 0.4942\, CSF^{-0.059}$$

D_* is the dimensionless particle size, which is calculated using:

$$D_* = D_g\left(\frac{g}{\nu^2}\frac{\rho_p - \rho_f}{\rho_f}\right)^{\frac{1}{3}}$$

where D_g is a reference diameter that includes the influence of particle shape:

$$\frac{D_g}{a} = (CSF)^{0.34}\left(\frac{b}{a}\right)^{0.5}$$

$$CSF = \frac{c}{\sqrt{ab}}$$

Where CSF is the Corey shape factor, a, b, and c are the longest, intermediate, and shortest dimension of the particle respectively (m), g is the gravitational acceleration (m/s^2), ν is the kinematic viscosity (m^2/s), ρ_p is the particle density (kg/m^3) and ρ_f is the fluid density (kg/m^3).

The dimensionless settling velocity W_* can then be used to calculate the actual settling velocity using the following equation:

$$W_* = \frac{w}{\left[\left(\frac{\rho_p - \rho_f}{\rho_f}\right) g\nu\right]^{\frac{1}{3}}}$$

Implementation:

4. Read in the mP dataset from Van Melkebeke *et al*[8].
1. Calculate additional required particle properties:
   a. Corey Shape factor (dimensionless): $CSF = \frac{c}{\sqrt{ab}}$
   b. Relative density (dimensinless): $\rho_{rel} = \frac{\rho_{mP} - \rho_w}{\rho_w}$
2. For each particle:
   a. Calculate the reference diameter (dimensionless): $\frac{D_g}{a} = (CSF)^{0.34}\left(\frac{b}{a}\right)^{0.5}$
   b. Calculate the dimensionless particle size: $D_* = D_g\left(\frac{g}{\nu^2}\frac{\rho_p - \rho_f}{\rho_f}\right)^{\frac{1}{3}}$
   c. Calculate the coefficient E (dimensionless): $E = \ a\left(\frac{a^2+b^2+c^2}{3}\right)^{-\frac{1}{2}}$
   d. Calculate the coefficient C_1 (dimensionless): $C_1 = 18\, E^{-0.38}$
   e. Calculate the coefficient C_2 (dimensionless): $C_2 = 0.3708\, CSF^{-0.1602}$
   f. Calculate the exponent n (dimensionless): $n = 0.4942\, CSF^{-0.059}$
   g. Calculate the dimensionless settling velocity $W_* = \frac{{D_*}^2}{C_1 + \left(0.75 C_2 {D_*}^3\right)^n}$
   h. Calculate the terminal settling velocity (m/s): $w = \ W_*\left[\left(\frac{\rho_p - \rho_f}{\rho_f}\right) g\nu\right]^{\frac{1}{3}}$

SI 8: Procedure to implement the implicit model by Yu *et al.* (2022)[11]

Description

The expression proposed by Yu *et al.,*[11] to calculate drag coefficient Cd is defined as:

$$C_d = \frac{C_{d,s}}{\left(d_*^{\beta_1} \Phi^{d_*^{\beta_2}} CSF^{d_*^{\beta_3}}\right)^{\beta_4}}$$

Where β1, β2, β3 and β4 are constants (β1=-0.25, β2=0.03, β3=0.33 and β4= 0.25), Φ is the particle sphericity, CSF is the Corey Shape Factor $CSF = \frac{c}{\sqrt{ab}}$ where a, b, and c are the longest, intermediate, and shortest dimension of the particle respectively (m) and $C_{d,s}$ is the drag coefficient for spherical particles proposed by Cheng (2009):

$$C_{d,s} = \frac{432}{d_*^3}(1 + 0.022d_*^3)^{0.54} + 0.47[1 - \exp(-0.15d_*^{0.45})]$$

The settling velocity of irregularly shaped particles is then defined as:

$$w_s = \left(\nu g \frac{\rho_s - \rho_f}{\rho_f}\right)^{\frac{1}{3}} \sqrt{\frac{4d_*}{3C_d}}$$

Where ν is the fluid kinematic viscosity (m^2/s), ρ_s is the particle density (kg/m^3) and ρ_f is the fluid density (kg/m^3).

The dimensionless particle diameter is defined as:

$$d_* = \left[\frac{(\rho_p - \rho_f)g}{\rho_f \nu^2}\right]^{\frac{1}{3}} d_n$$

where d_n is the volume equivalent spherical diameter

Implementation:

1. Read in the mP dataset from Van Melkebeke *et al*[8].
2. For each particle:
    a. Calculate the Corey Shape factor (dimensionless): $CSF = \frac{c}{\sqrt{ab}}$
    b. Calculate the dimensionless particle size: $d_* = \left[\frac{(\rho_p - \rho_f)g}{\rho_f \nu^2}\right]^{\frac{1}{3}} d_n$
    c. Calculate the drag coefficient for spherical particles: $C_{d,s} = \frac{432}{d_*^3}(1 + 0.022d_*^3)^{0.54} + 0.47[1 - \exp(-0.15d_*^{0.45})]$
    d. Calculate the drag coefficient C_d (dimensionless): $C_d = \frac{C_{d,s}}{\left(d_*^{\beta_1} \Phi^{d_*^{\beta_2}} CSF^{d_*^{\beta_3}}\right)^{\beta_4}}$
    e. Calculate the terminal settling velocity (m/s): $w_s = \left(\nu g \frac{\rho_s - \rho_f}{\rho_f}\right)^{\frac{1}{3}} \sqrt{\frac{4d_*}{3C_d}}$

SI 9: Results of all morphologies for each implicit model tested during the evaluation of the empirical drag models.

a) Stokes' Model[1]

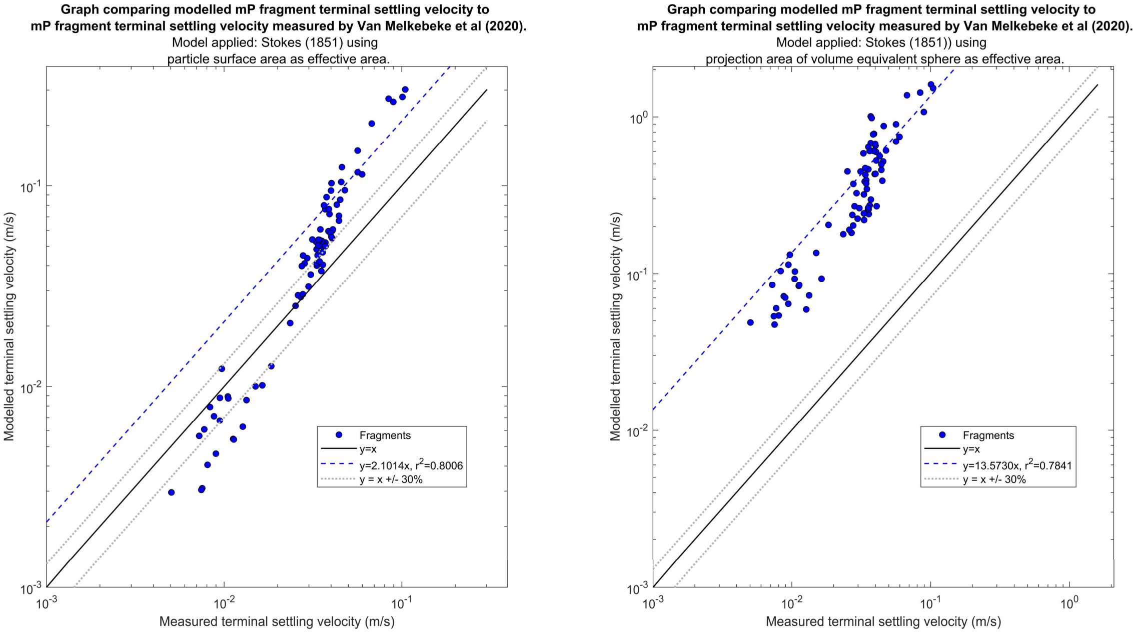

Figure S1: Measured settling velocity of fragment mP particles in the dataset from Van Melkebeke et al.[8] compared to the terminal settling velocity predicted using Stokes' model[1]. The figure on the left shows the results when particle surface area was used as the effective area during the calculation of the drag force and the figure on the right shows the results when the projection area was used as the effective area during the calculation of the drag force. The solid line indicates the ideal fit where estimated terminal settling velocity equals measured terminal settling velocity and the dotted lines indicate estimated terminal settling velocity equals ±30% of measured terminal settling velocity. The dashed line indicates the best fit line in the form y=mx that was obtained using linear regression.

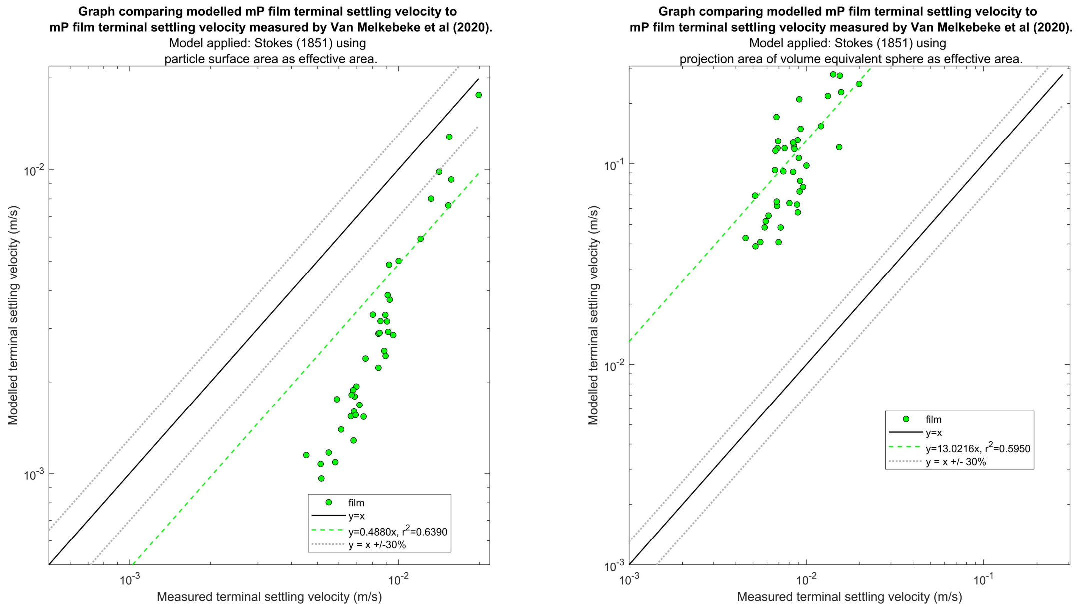

Figure S2: Measured settling velocity of film mP particles in the dataset from Van Melkebeke et al.[8] compared to the terminal settling velocity predicted using Stokes' model[1]. The figure on the left shows the results when particle surface area was used as the effective area during the calculation of the drag force and the figure on the right shows the results when the projection area was used as the effective area during the calculation of the drag force. The solid line indicates the ideal fit where estimated terminal settling velocity equals measured terminal settling velocity and the dotted lines indicate estimated terminal settling velocity equals ±30% of measured terminal settling velocity. The dashed line indicates the best fit line in the form y=mx that was obtained using linear regression.

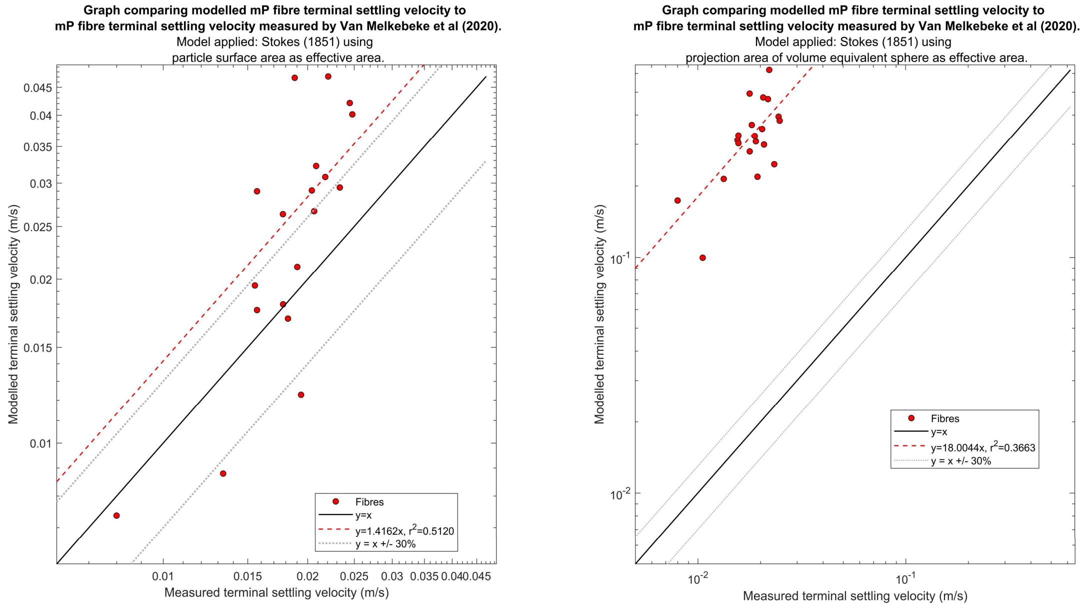

Figure S3: Measured settling velocity of fibrous mP particles in the dataset from Van Melkebeke et al.[8] compared to the terminal settling velocity predicted using Stokes' model[1]. The figure on the left shows the results when particle surface area was used as the effective area during the calculation of the drag force and the figure on the right shows the results when the projection area was used as the effective area during the calculation of the drag force. The solid line indicates the ideal fit where estimated terminal settling velocity equals measured terminal settling velocity and the dotted lines indicate estimated terminal settling velocity equals ±30% of measured terminal settling velocity. The dashed line indicates the best fit line in the form y=mx that was obtained using linear regression.

b) Bagheri and Bonadonna's model[4]

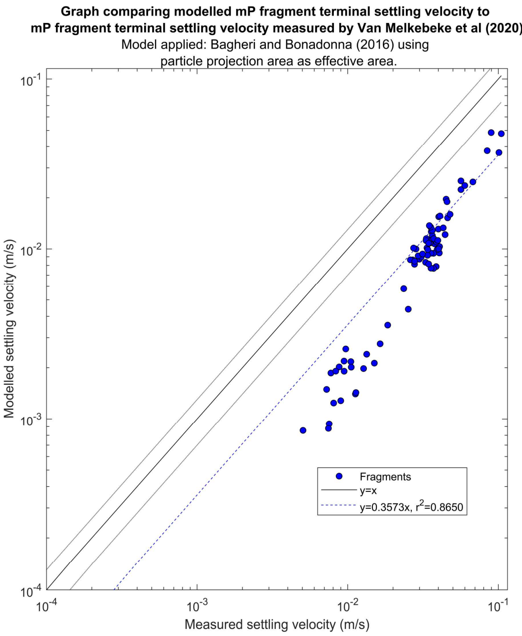

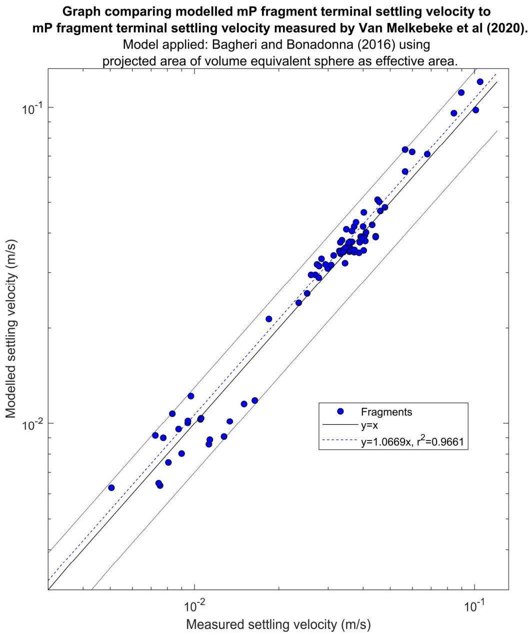

Figure S4: Measured settling velocity of fragment mP particles in the dataset from Van Melkebeke *et al*.[8] compared to the terminal settling velocity predicted using Bagheri and Bonadonna's model[4]. The figure on the left shows the results when particle surface area was used as the effective area during the calculation of the drag force and the figure on the right shows the results when the projection area was used as the effective area during the calculation of the drag force. The solid line indicates the ideal fit where estimated terminal settling velocity equals measured terminal settling velocity and the dotted lines indicate estimated terminal settling velocity equals ±30% of measured terminal settling velocity. The dashed line indicates the best fit line in the form y=mx that was obtained using linear regression.

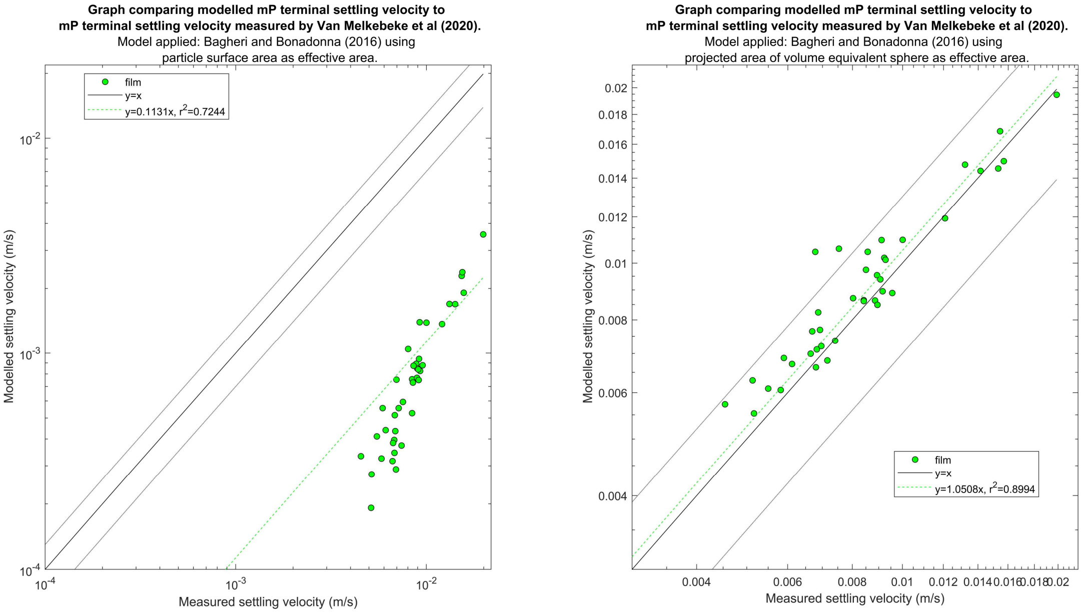

Figure S5: Measured settling velocity of film mP particles in the dataset from Van Melkebeke et al.[8] compared to the terminal settling velocity predicted using Bagheri and Bonadonna's model[4]. The figure on the left shows the results when particle surface area was used as the effective area during the calculation of the drag force and the figure on the right shows the results when the projection area was used as the effective area during the calculation of the drag force. The solid line indicates the ideal fit where estimated terminal settling velocity equals measured terminal settling velocity and the dotted lines indicate estimated terminal settling velocity equals ±30% of measured terminal settling velocity. The dashed line indicates the best fit line in the form y=mx that was obtained using linear regression.

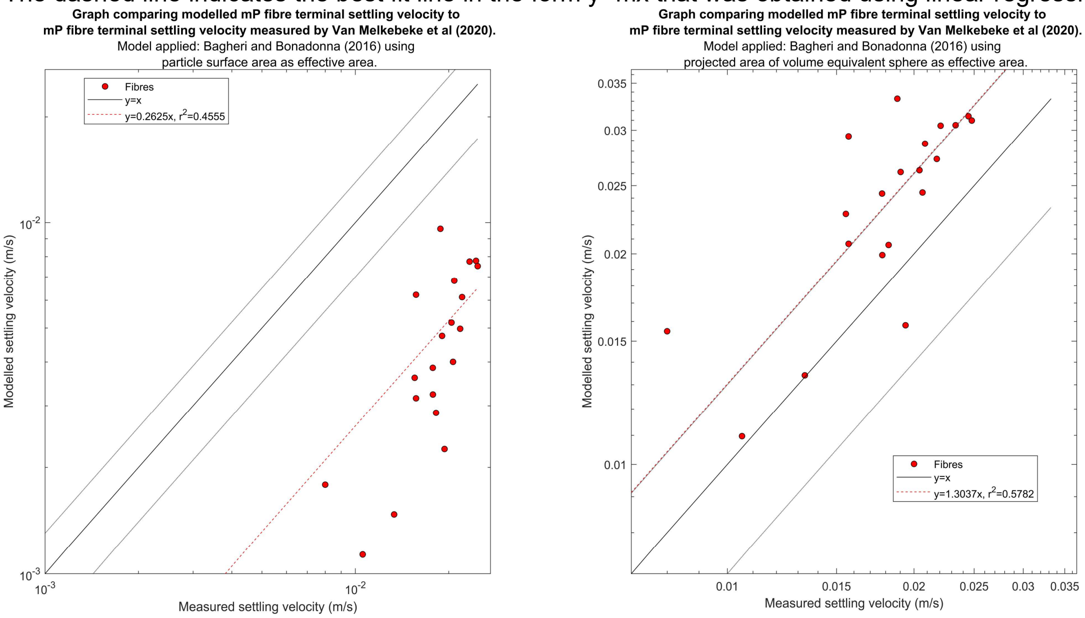

Figure S6: Measured settling velocity of fibrous mP particles in the dataset from Van Melkebeke et al.[8] compared to the terminal settling velocity predicted using Bagheri and Bonadonna's model[4]. The figure on the left shows the results when particle surface area was used as the effective area during the calculation of the drag force and the figure on the right shows the results when the projection area was used as the effective area during the calculation of the drag force. The solid line indicates the ideal fit where estimated terminal settling velocity equals measured terminal settling velocity and the dotted lines indicate estimated terminal settling velocity equals ±30% of measured terminal settling velocity. The dashed line indicates the best fit line in the form y=mx that was obtained using linear regression.

c) Dioguardi et al.*'s model*[5]

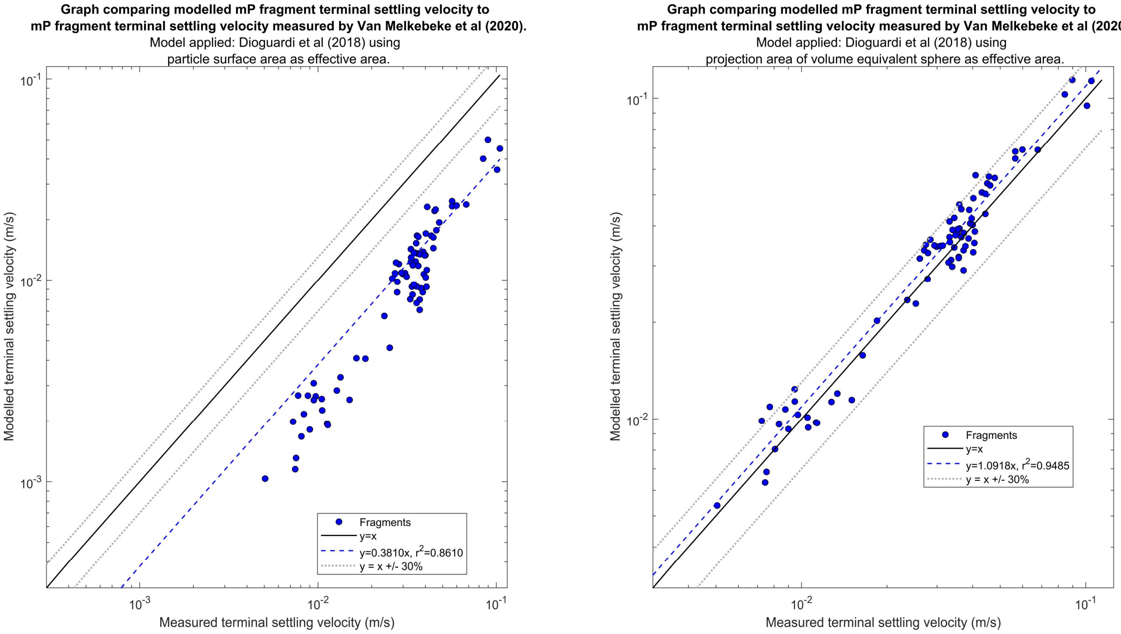

Figure S7: Measured settling velocity of fragment mP particles in the dataset from Van Melkebeke *et al.*[8] compared to the terminal settling velocity predicted using Dioguardi *et al.*'s model[5]. The figure on the left shows the results when particle surface area was used as the effective area during the calculation of the drag force and the figure on the right shows the results when the projection area was used as the effective area during the calculation of the drag force. The solid line indicates the ideal fit where estimated terminal settling velocity equals measured terminal settling velocity and the dotted lines indicate estimated terminal settling velocity equals ±30% of measured terminal settling velocity. The dashed line indicates the best fit line in the form y=mx that was obtained using linear regression.

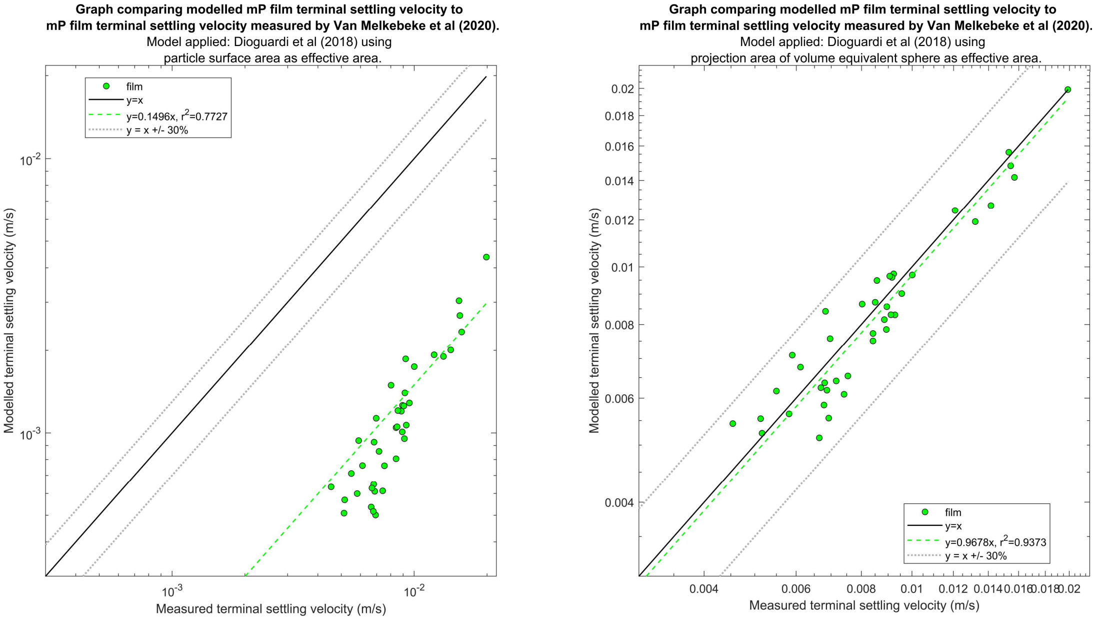

Figure S8: Measured settling velocity of film mP particles in the dataset from Van Melkebeke et al.[8] compared to the terminal settling velocity predicted using Dioguardi et al.'s model[5]. The figure on the left shows the results when particle surface area was used as the effective area during the calculation of the drag force and the figure on the right shows the results when the projection area was used as the effective area during the calculation of the drag force. The solid line indicates the ideal fit where estimated terminal settling velocity equals measured terminal settling velocity and the dotted lines indicate estimated terminal settling velocity equals ±30% of measured terminal settling velocity. The dashed line indicates the best fit line in the form y=mx that was obtained using linear regression.

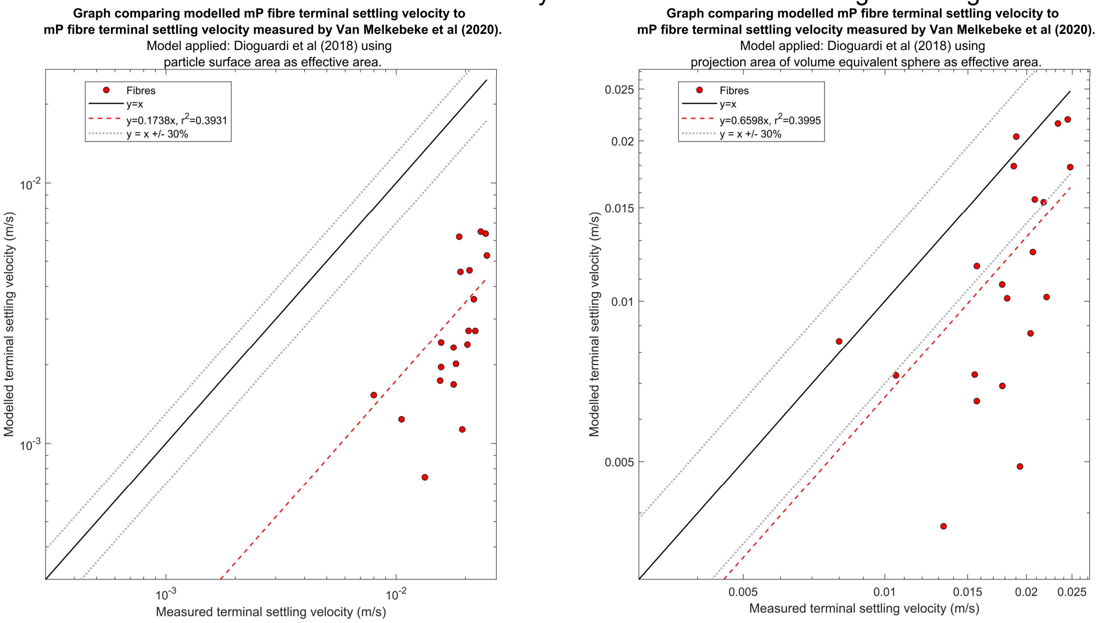

Figure S9: Measured settling velocity of fibrous mP particles in the dataset from Van Melkebeke et al.[8] compared to the terminal settling velocity predicted using Dioguardi et al.'s model[5]. The figure on the left shows the results when particle surface area was used as the effective area during the calculation of the drag force and the figure on the right shows the results when the projection area was used as the effective area during the calculation of the drag force. The solid line indicates the ideal fit where estimated terminal settling velocity equals measured terminal settling velocity and the dotted lines indicate estimated terminal settling velocity equals ±30% of measured terminal settling velocity. The dashed line indicates the best fit line in the form y=mx that was obtained using linear regression.

d) Zhang and Choi's model[7]

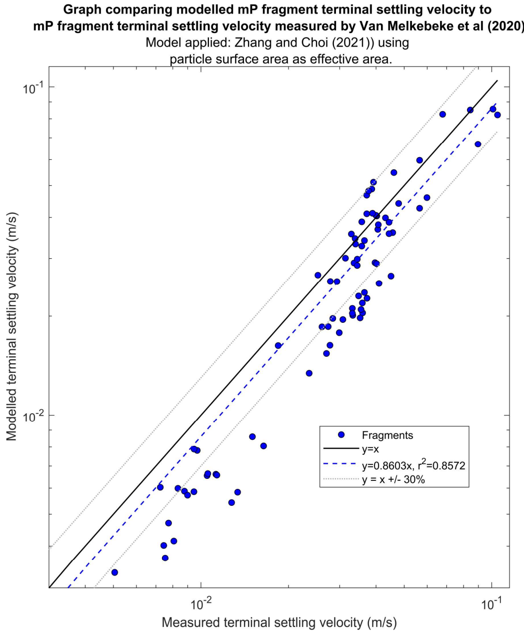

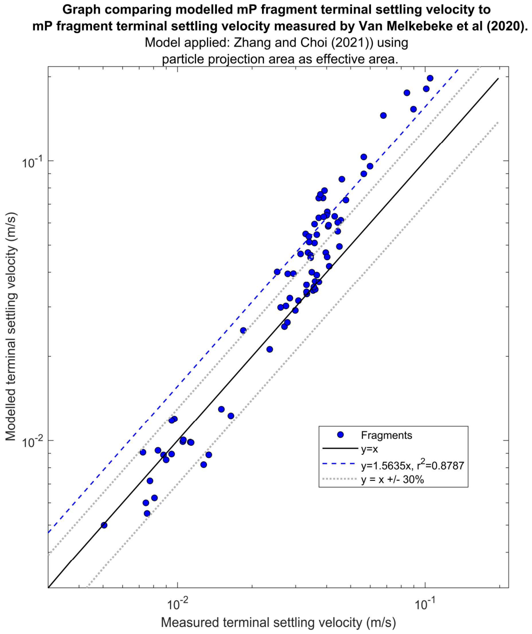

Figure S10: Measured settling velocity of fragment mP particles in the dataset from Van Melkebeke *et al.*[8] compared to the terminal settling velocity predicted using Zhang and Choi's model[7]. The figure on the left shows the results when particle surface area was used as the effective area during the calculation of the drag force and the figure on the right shows the results when the projection area was used as the effective area during the calculation of the drag force The solid line indicates the ideal fit where estimated terminal settling velocity equals measured terminal settling velocity and the dotted lines indicate estimated terminal settling velocity equals ±30% of measured terminal settling velocity. The dashed line indicates the best fit line in the form y=mx that was obtained using linear regression.

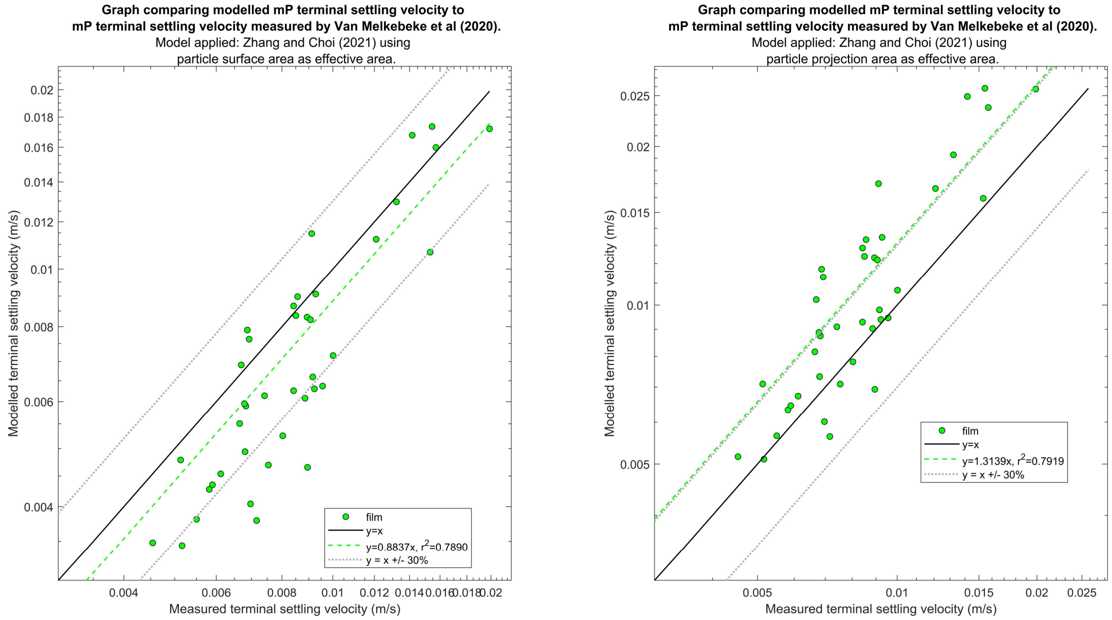

Figure S11: Measured settling velocity of film mP particles in the dataset from Van Melkebeke et al.[8] compared to the terminal settling velocity predicted using Zhang and Choi's model[7]. The figure on the left shows the results when particle surface area was used as the effective area during the calculation of the drag force and the figure on the right shows the results when the projection area was used as the effective area during the calculation of the drag force. The solid line indicates the ideal fit where estimated terminal settling velocity equals measured terminal settling velocity and the dotted lines indicate estimated terminal settling velocity equals ±30% of measured terminal settling velocity. The dashed line indicates the best fit line in the form y=mx that was obtained using linear regression.

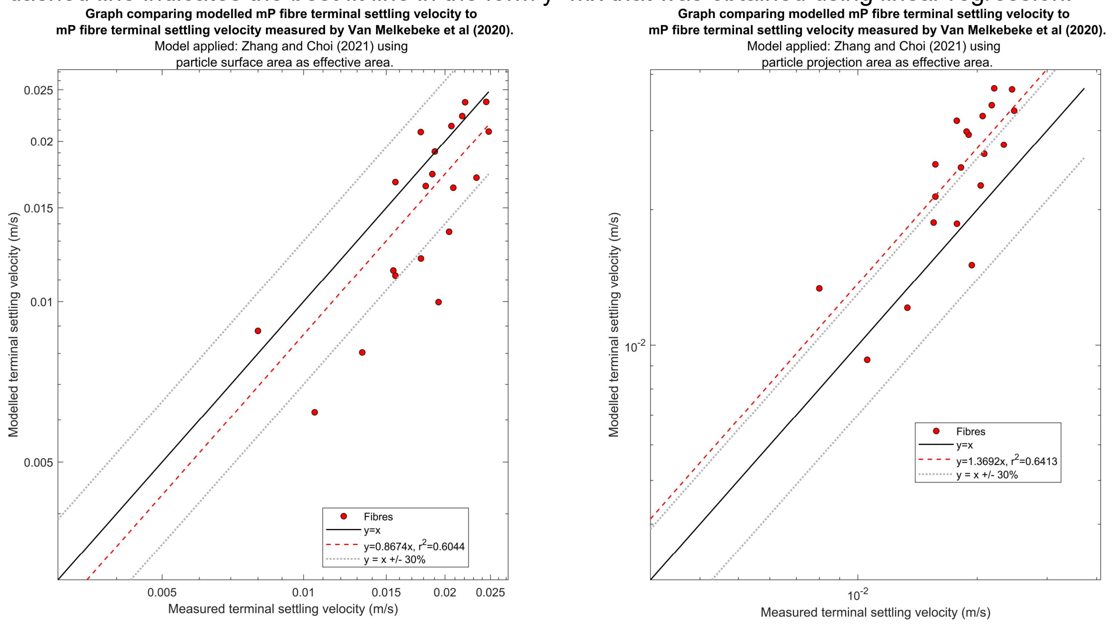

Figure S12: Measured settling velocity of fibrous mP particles in the dataset from Van Melkebeke et al.[8] compared to the terminal settling velocity predicted using Zhang and Choi's model[7]. The figure on the left shows the results when particle surface area was used as the effective area during the calculation of the drag force and the figure on the right shows the results when the projection area was used as the effective area during the calculation of the drag force. The solid line indicates the ideal fit where estimated terminal settling velocity equals measured terminal settling velocity and the dotted lines indicate estimated terminal settling velocity equals ±30% of measured terminal settling velocity. The dashed line indicates the best fit line in the form y=mx that was obtained using linear regression.

SI 10: Results of all morphologies for each explicit model tested during the evaluation of the empirical drag models.

a) ***Dietrich's model***[9]

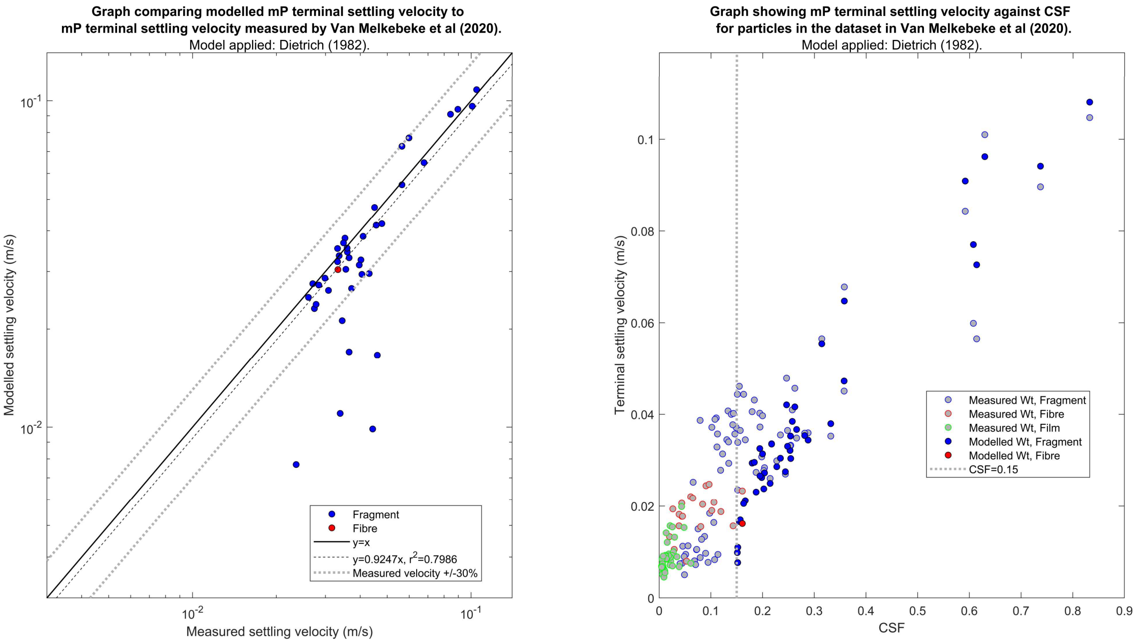

Figure S13: The figure on the right shows a comparison of measured terminal settling velocity to the terminal settling velocity calculated using Dietrich's model[9]. The solid line indicates the ideal fit where estimated terminal settling velocity equals measured terminal settling velocity and the dotted lines indicate estimated terminal settling velocity equals ±30% of measured terminal settling velocity. The dashed line indicates the best fit line in the form y=mx that was obtained using linear regression.The figure on the right shows the terminal settling velocity against the Corey Shape Factor (CSF) to highlight that the model is not valid for particles which have a CSF of less than 0.15.

***b) Francalanci* et al*.’s model*[10]**

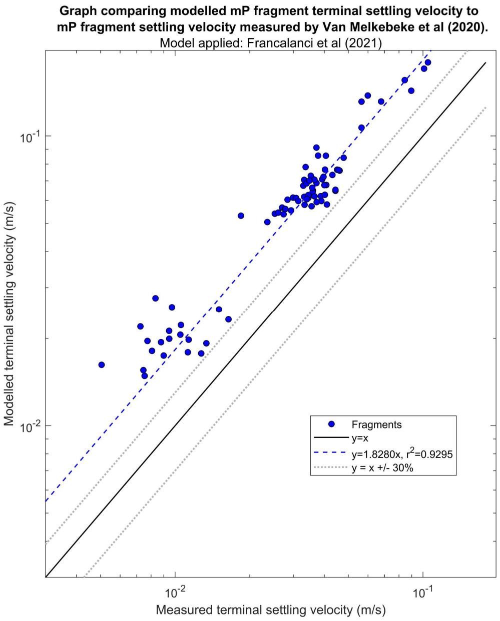

Figure S14: Measured settling velocity of fragment mP particles in the dataset from Van Melkebeke *et al.*[8] compared to the terminal settling velocity predicted using the model by Francalanci *et al.*[10]. The solid line indicates the ideal fit where estimated terminal settling velocity equals measured terminal settling velocity and the dotted lines indicate estimated terminal settling velocity equals ±30% of measured terminal settling velocity. The dashed line indicates the best fit line in the form y=mx that was obtained using linear regression.

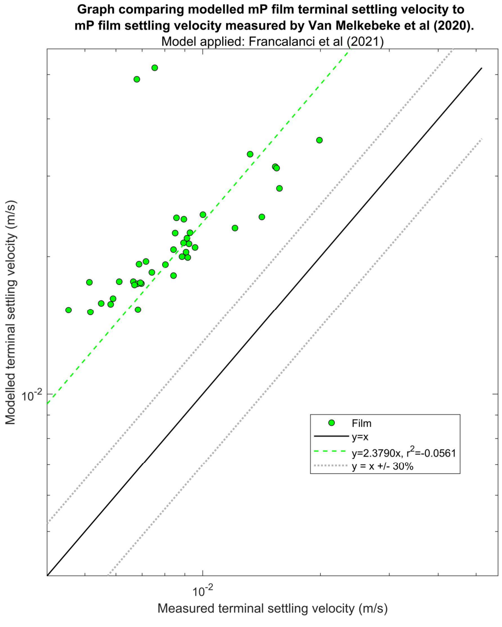

Figure S15: Measured settling velocity of film mP particles in the dataset from Van Melkebeke *et al.*[8] compared to the terminal settling velocity predicted using the model by Francalanci *et al.*[10]. The solid line indicates the ideal fit where estimated terminal settling velocity equals measured terminal settling velocity and the dotted lines indicate estimated terminal settling velocity equals ±30% of measured terminal settling velocity. The dashed line indicates the best fit line in the form y=mx that was obtained using linear regression.

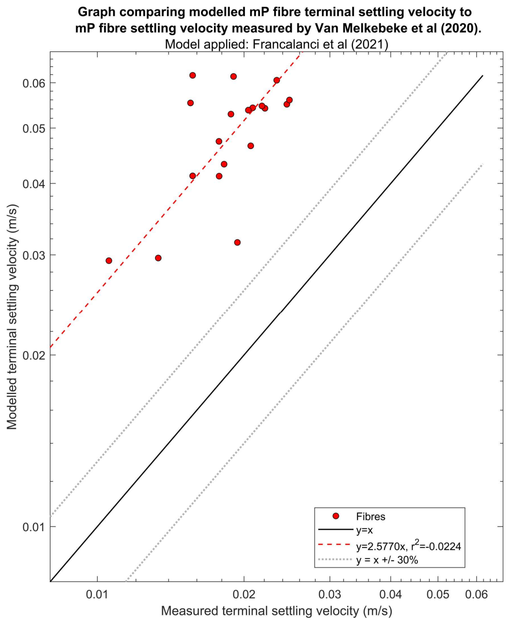

Figure S16: Measured settling velocity of film mP particles in the dataset from Van Melkebeke *et al.*[8] compared to the terminal settling velocity predicted using the model by Francalanci *et al.*[10]. The solid line indicates the ideal fit where estimated terminal settling velocity equals measured terminal settling velocity and the dotted lines indicate estimated terminal settling velocity equals ±30% of measured terminal settling velocity. The dashed line indicates the best fit line in the form y=mx that was obtained using linear regression.

c) Yu et al.*'s model*[11]

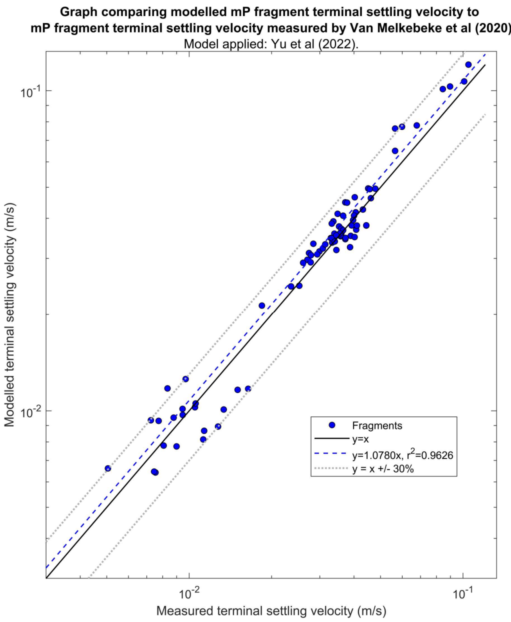

Figure S17: Measured settling velocity of fragment mP particles in the dataset from Van Melkebeke et al.[8] compared to the terminal settling velocity predicted using the model by Yu et al.[11]. The solid line indicates the ideal fit where estimated terminal settling velocity equals measured terminal settling velocity and the dotted lines indicate estimated terminal settling velocity equals ±30% of measured terminal settling velocity. The dashed line indicates the best fit line in the form y=mx that was obtained using linear regression.

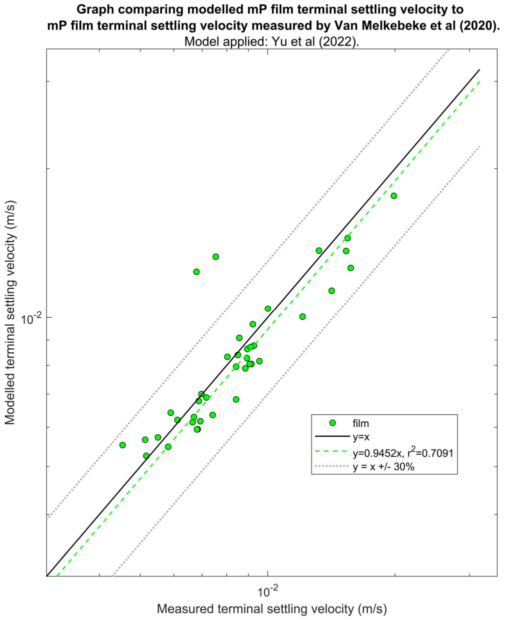

Figure S18: Measured settling velocity of film mP particles in the dataset from Van Melkebeke *et al.*[8] compared to the terminal settling velocity predicted using the model by Yu *et al.*[11]. The solid line indicates the ideal fit where estimated terminal settling velocity equals measured terminal settling velocity and the dotted lines indicate estimated terminal settling velocity equals ±30% of measured terminal settling velocity. The dashed line indicates the best fit line in the form y=mx that was obtained using linear regression.

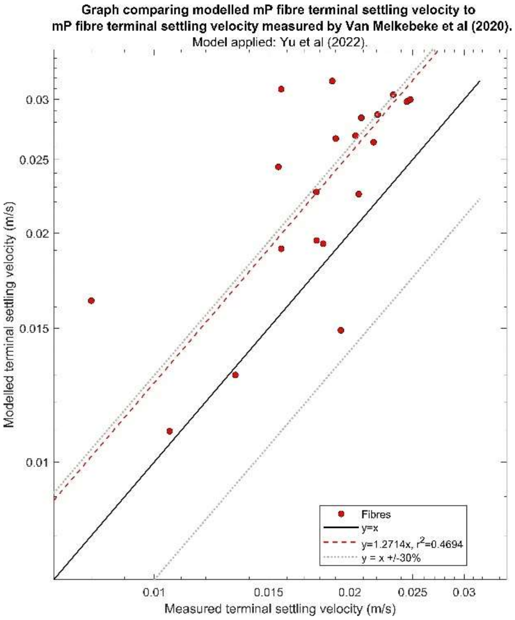

Figure S19: Measured settling velocity of fibrous mP particles in the dataset from Van Melkebeke *et al.*[8] compared to the terminal settling velocity predicted using the model by Yu *et al.*[11]. The solid line indicates the ideal fit where estimated terminal settling velocity equals measured terminal settling velocity and the dotted lines indicate estimated terminal settling velocity equals ±30% of measured terminal settling velocity. The dashed line indicates the best fit line in the form y=mx that was obtained using linear regression.

SI 11: Results for each model tested during the evaluation of the impact of the choice of initial velocity on the result of the implicit models.

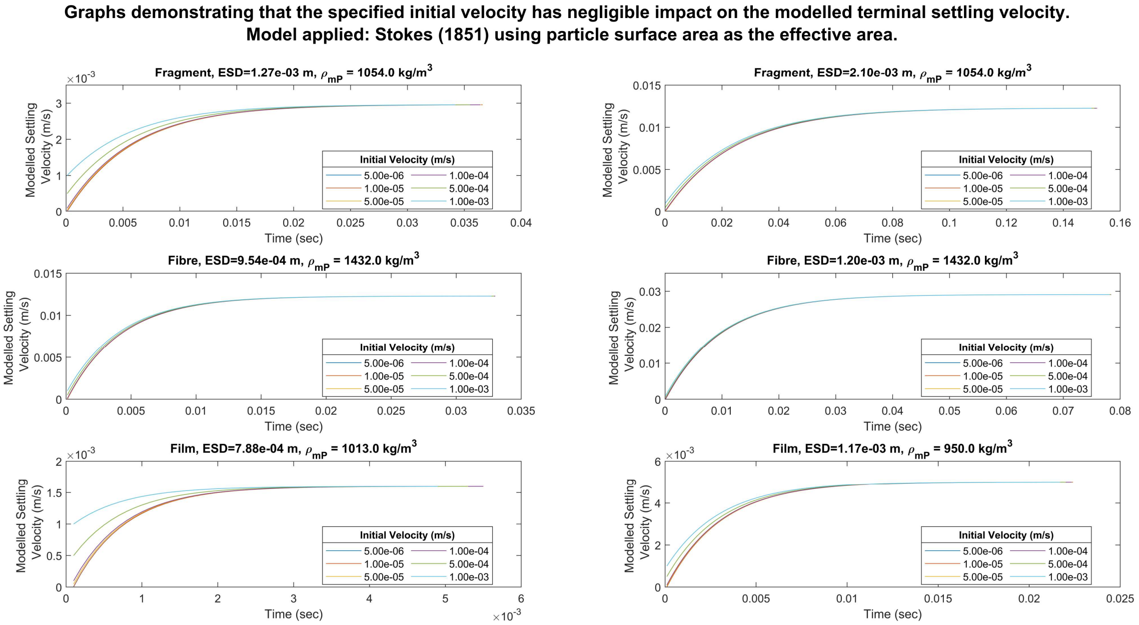

Figure S20: The impact of the choice of initial velocity on the modelled settling velocity when using Stokes' model[1] for six particles that were randomly extracted from the dataset by Van Melkebeke *et al.*[8].

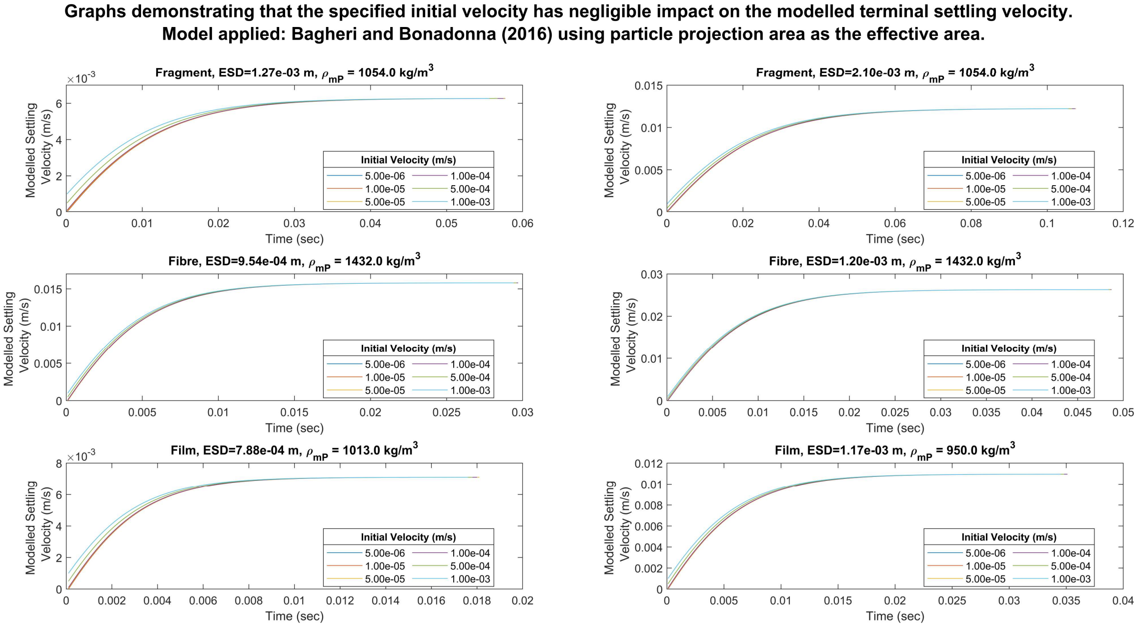

Figure S21: The impact of the choice of initial velocity on the modelled settling velocity when using Bagheri and Bonadonna's model[4] for six particles that were randomly extracted from the dataset by Van Melkebeke *et al.*[8].

Graphs demonstrating that the specified initial velocity has negligible impact on the modelled terminal settling velocity.
Model applied: Dioguardi et al (2018) using particle projection area as the effective area.

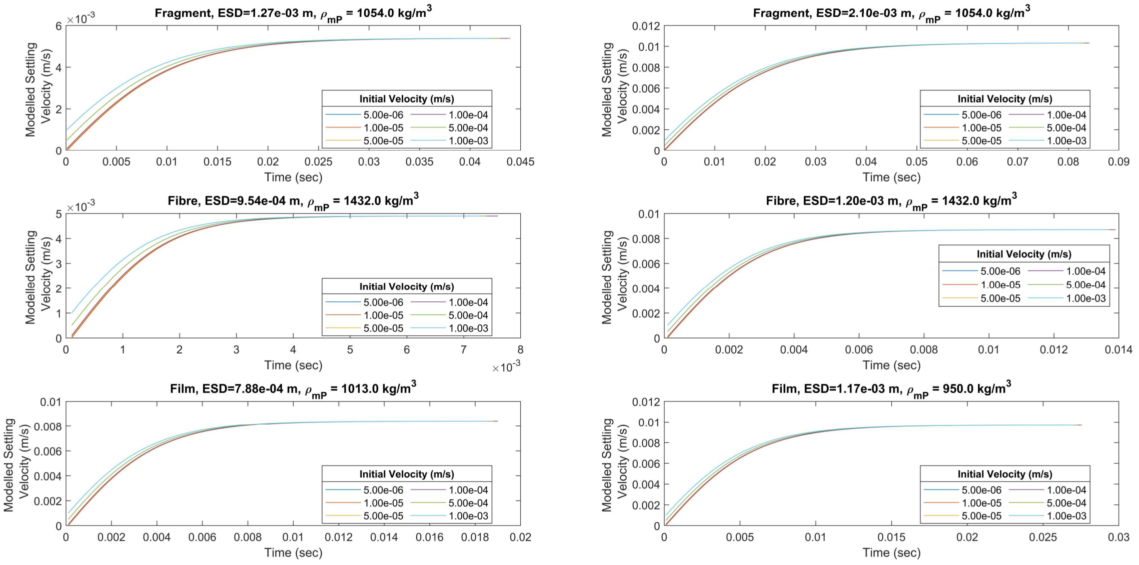

Figure S22: The impact of the choice of initial velocity on the modelled settling velocity when using Dioguardi *et al*.'s model[5] for six particles that were randomly extracted from the dataset by Van Melkebeke *et al*.[8].

Graphs demonstrating that the specified initial velocity has negligible impact on the modelled terminal settling velocity.
Model applied: Zhang and Choi (2021) using particle projection area as the effective area.

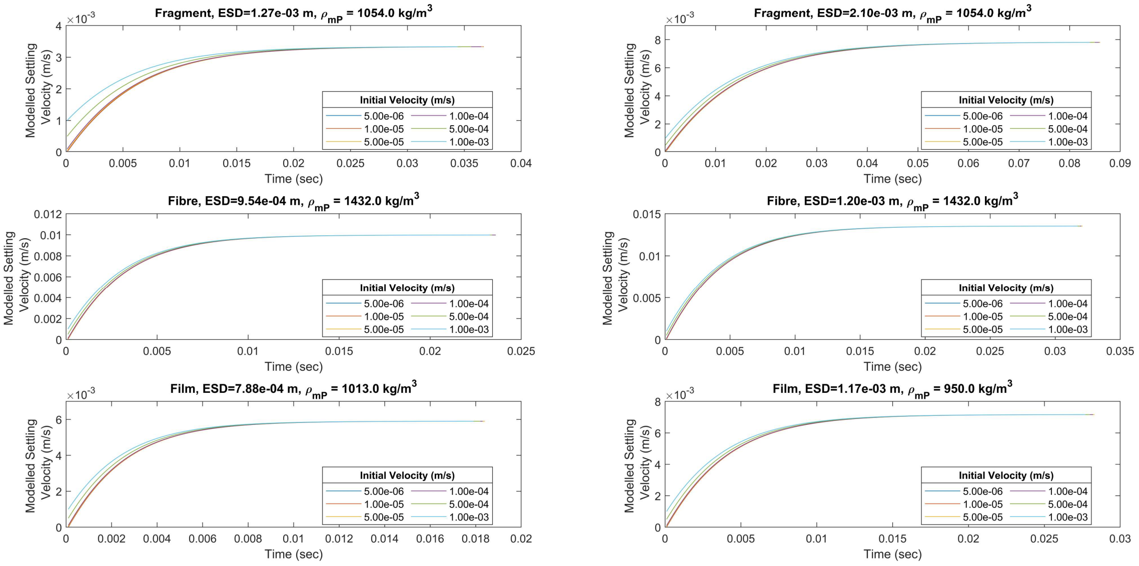

Figure S23: The impact of the choice of initial velocity on the modelled settling velocity of six particles when using Zhang and Choi's model[7] and taking the particle projection area as the effective area in the calculation of the drag force that were randomly extracted from the dataset by Van Melkebeke *et al*.[8].

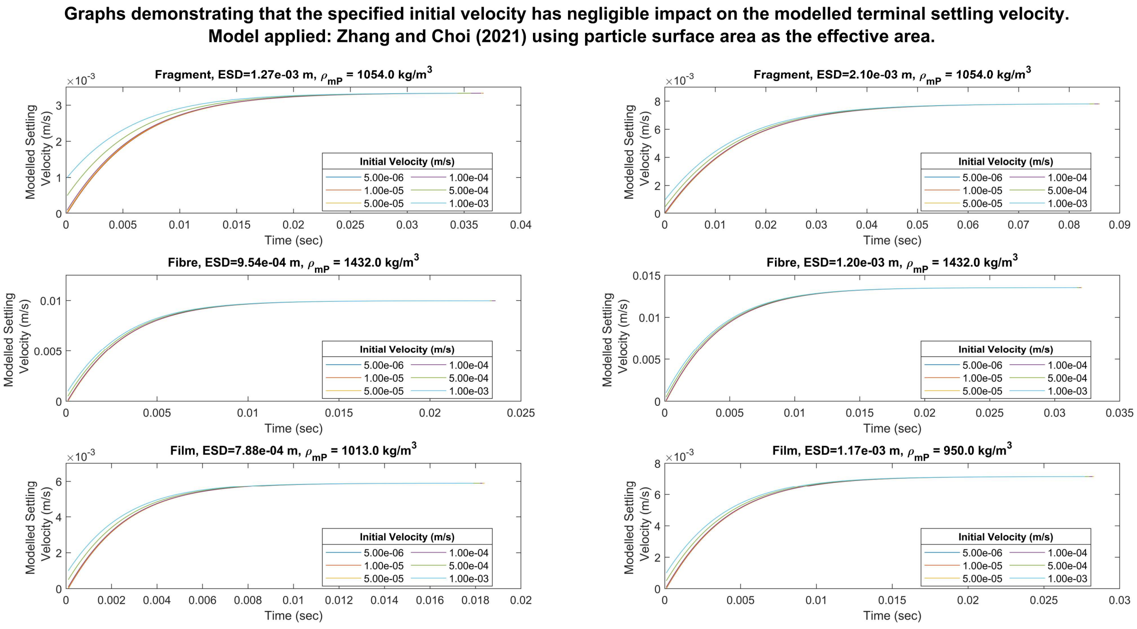

Figure S24: The impact of the choice of initial velocity on the modelled settling velocity of six particles when using Zhang and Choi's model[7] and taking the particle surface area as the effective area in the calculation of the drag force that were randomly extracted from the dataset by Van Melkebeke *et al*.[8].

SI 12: Results for each model tested during the evaluation of the variation in the terminal settling velocity over the range of density in the ocean and the impact on the models of assuming a constant terminal settling velocity.

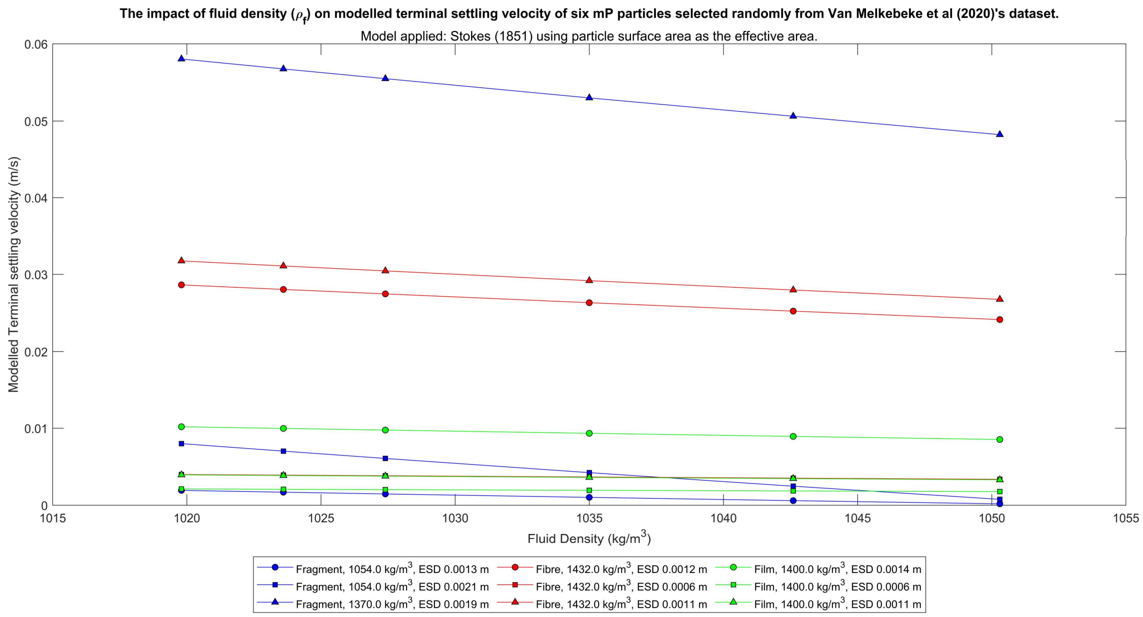

Figure S25: The influence of fluid density on the terminal settling velocity of six random particles using Stokes' model[1].

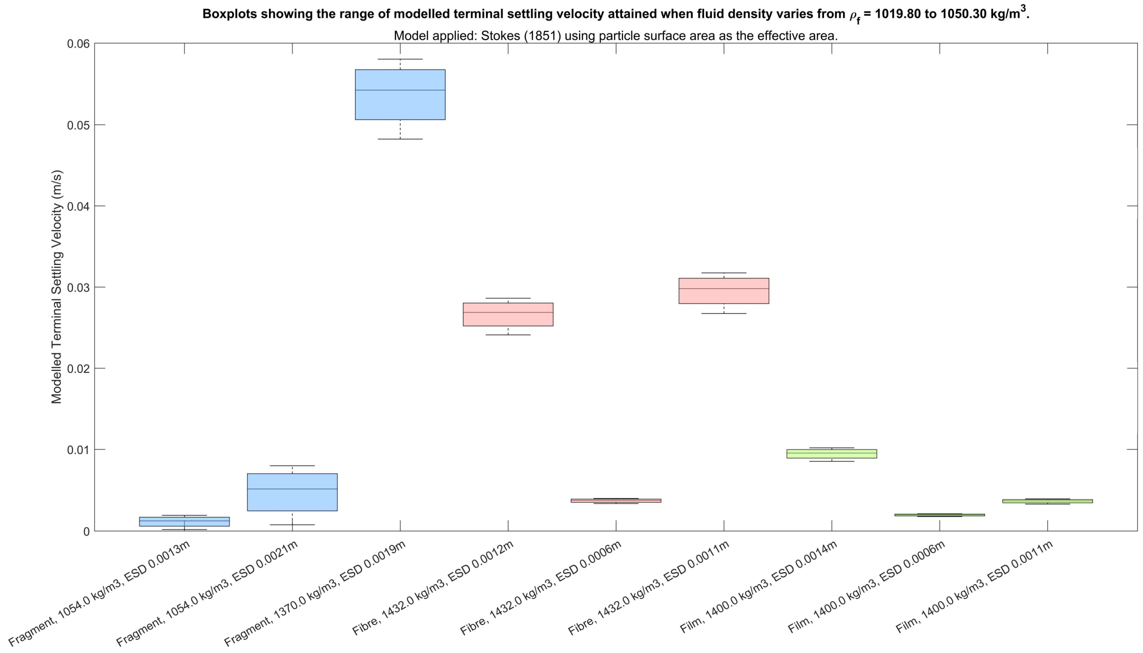

Figure S26: The range of settling velocity obtained for each of six random particles using Stokes' model[1] when the fluid density was varied from 1019 to 1050 kg/m^3..

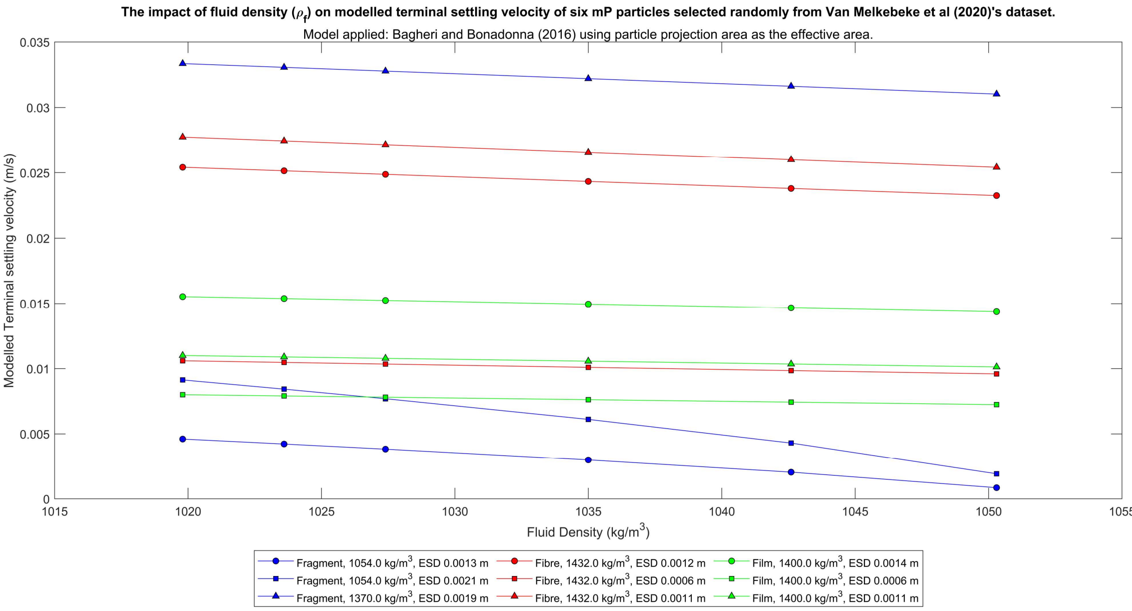

Figure S27: The influence of fluid density on the terminal settling velocity of six random particles using Bagheri and Bonadonna's model[4].

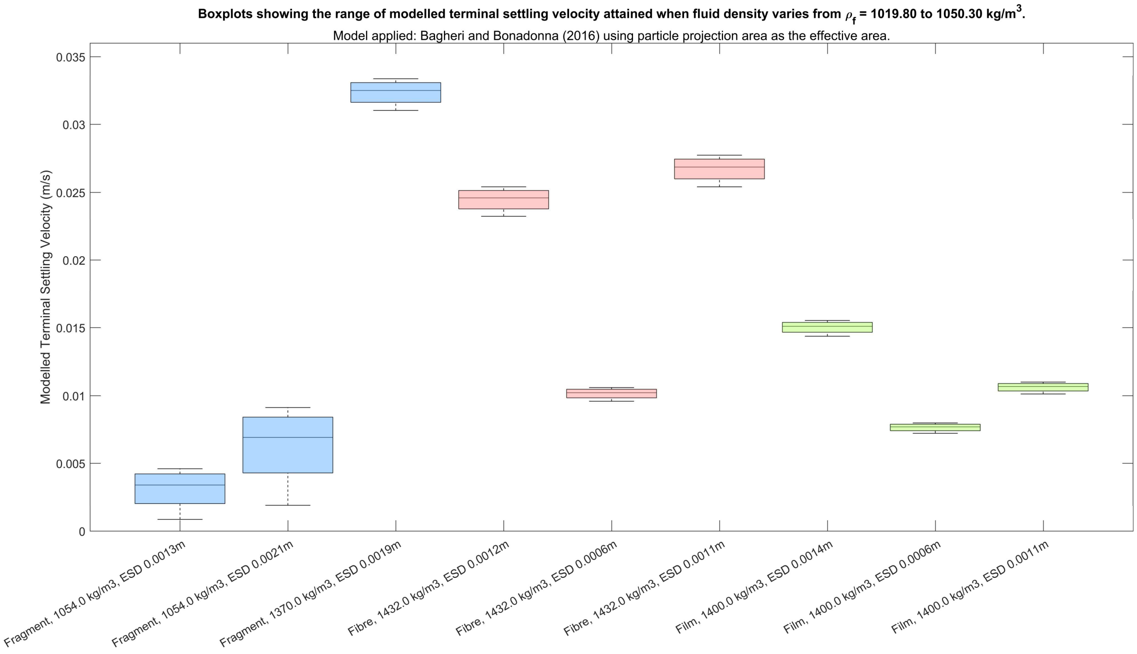

Figure S28: The range of settling velocity obtained for each of six random particles using Bagheri and Bonadonna's model[4] when the fluid density was varied from 1019 to 1050 kg/m^3.

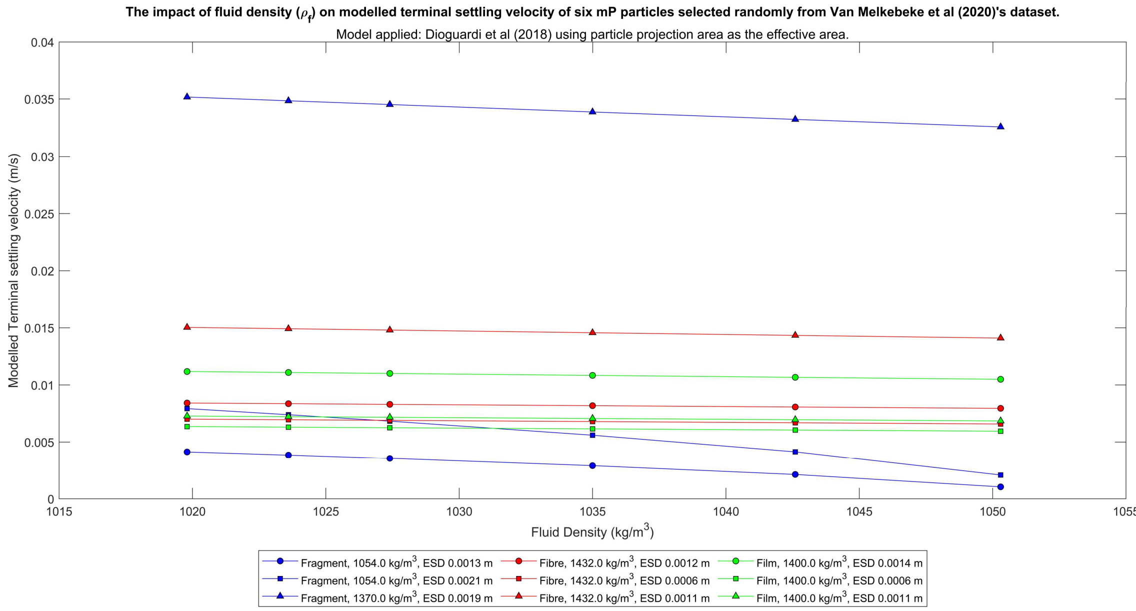

Figure S29: The influence of fluid density on the terminal settling velocity of six random particles using Dioguardi *et al.*'s model[5].

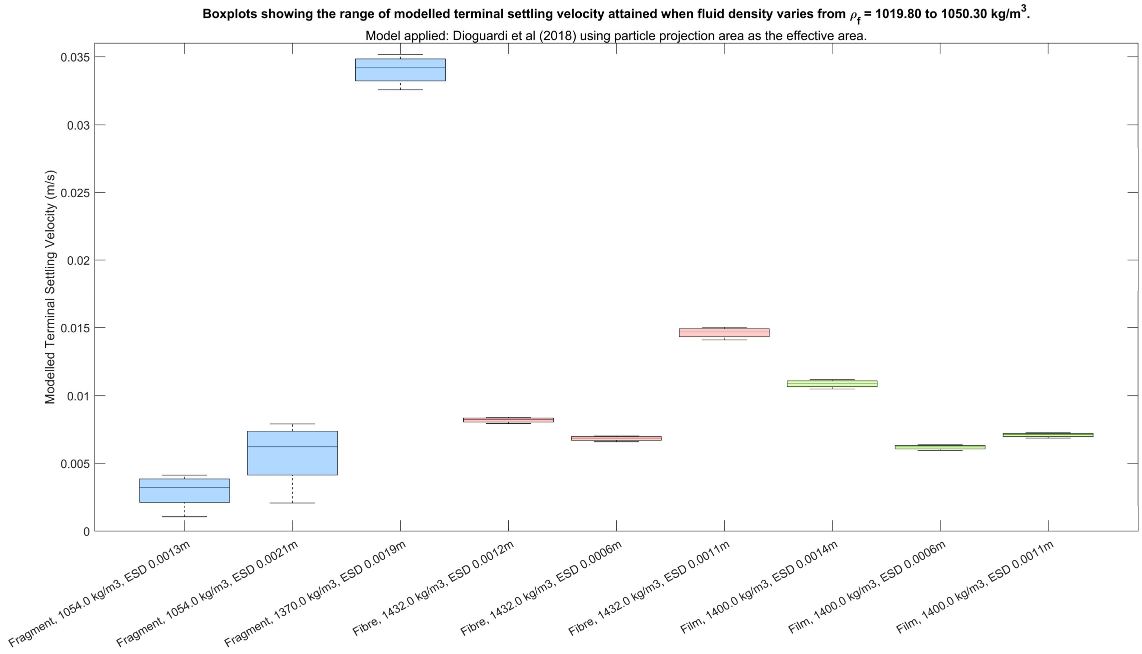

Figure S30: The range of settling velocity obtained for each of six random particles using Dioguardi *et al.*'s model[5] when the fluid density was varied from 1019 to 1050 kg/m^3.

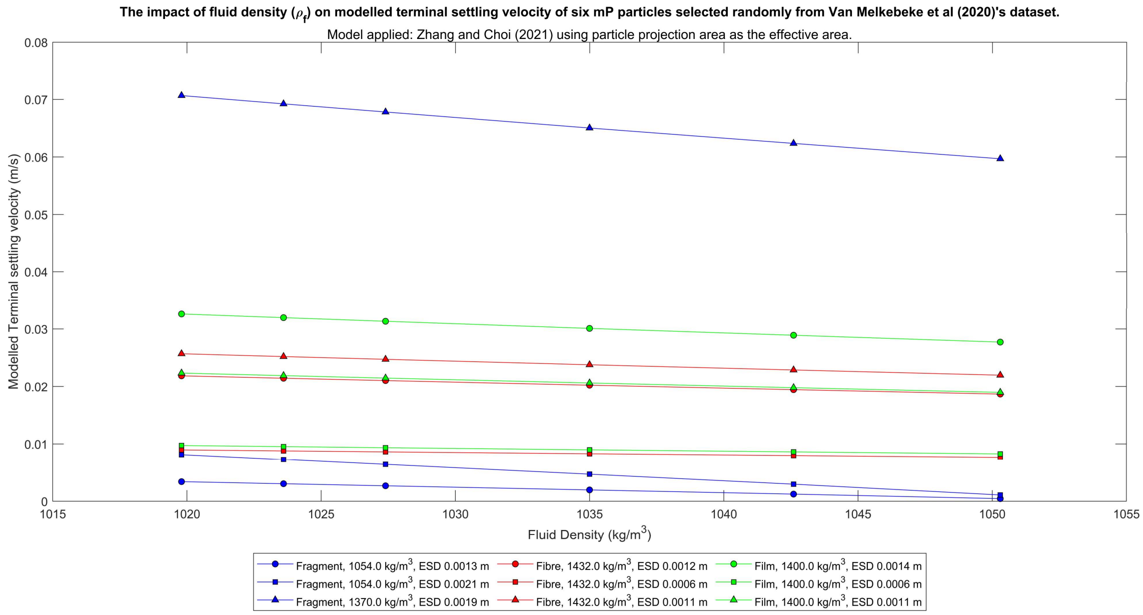

Figure S31: The influence of fluid density on the terminal settling velocity of six random particles using Zhang and Choi"s model[7] and taking the particle projected area as the effective area in the calculation of the drag force.

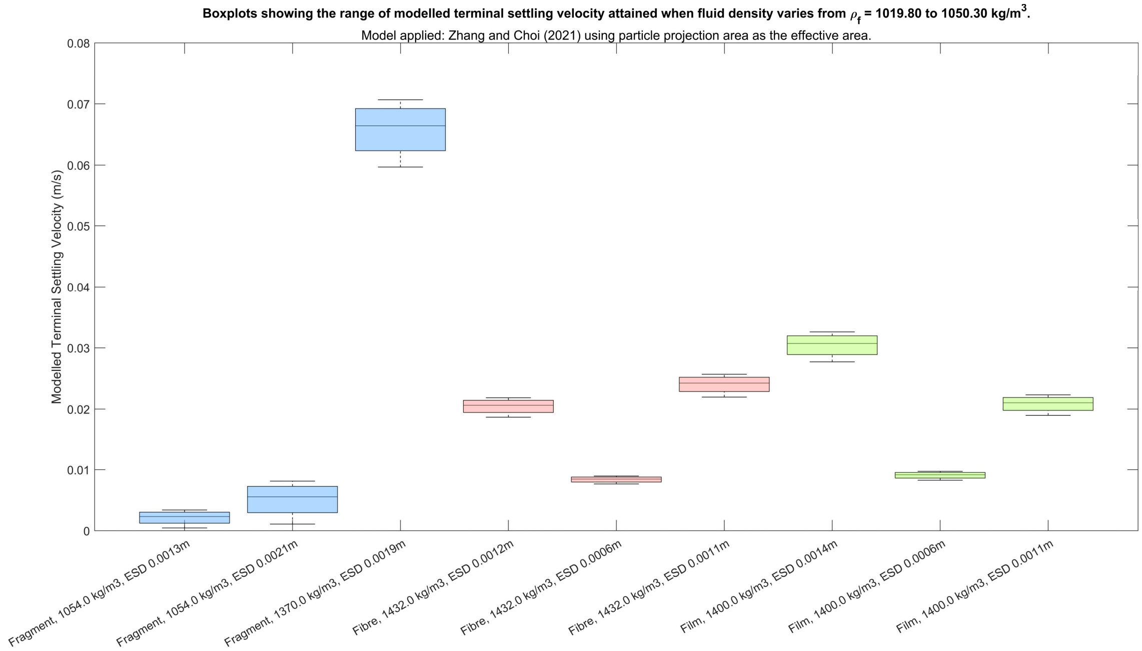

Figure S32: The range of settling velocity obtained for each of six random particles using Zhang and Choi"s model[7] and taking the particle projected area as the effective area in the calculation of the drag force when the fluid density was varied from 1019 to 1050 kg/m^3.

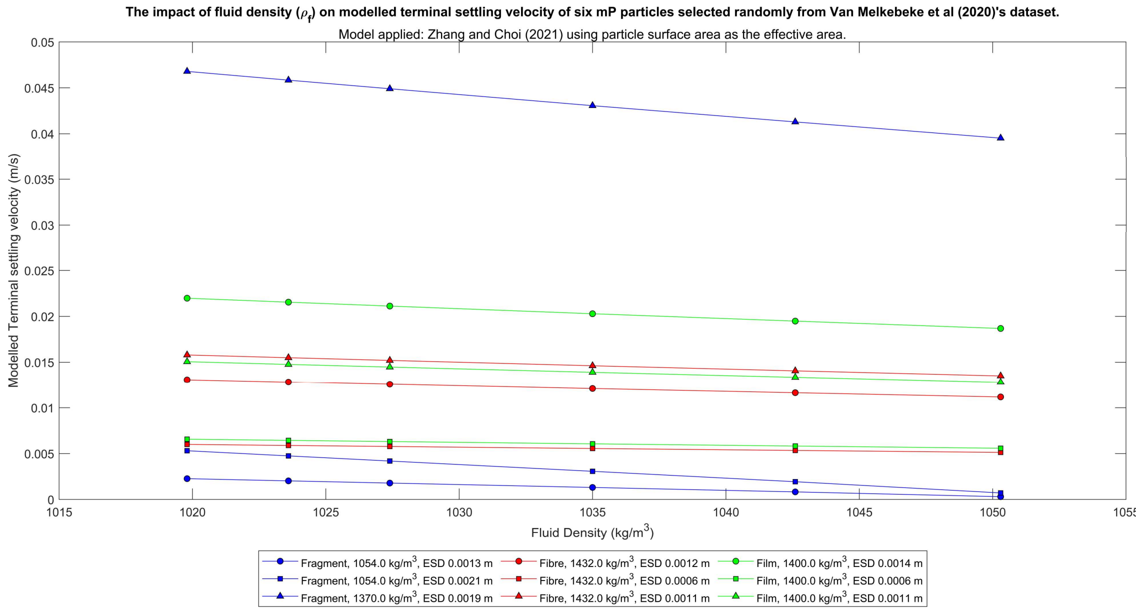

Figure S33: The influence of fluid density on the terminal settling velocity of six random particles using Zhang and Choi"s model[7] and taking the particle surface area as the effective area in the calculation of the drag force.

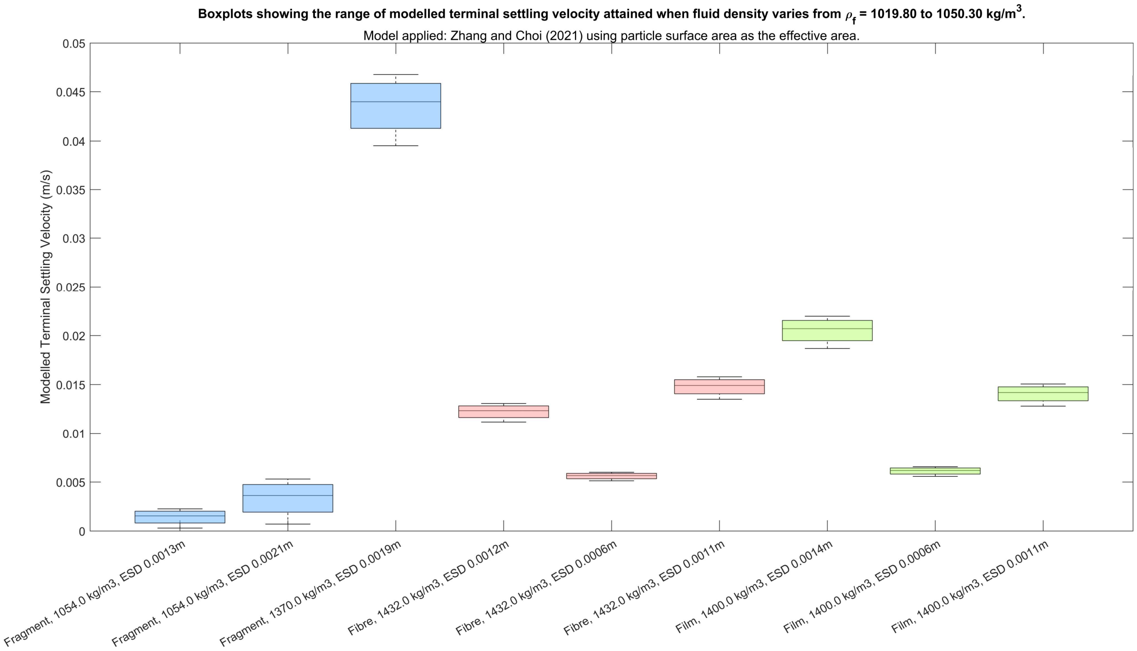

Figure S34: The range of settling velocity obtained for each of six random particles using Zhang and Choi"s model[7] and taking the particle surface area as the effective area in the calculation of the drag force when the fluid density was varied from 1019 to 1050 kg/m^3

.

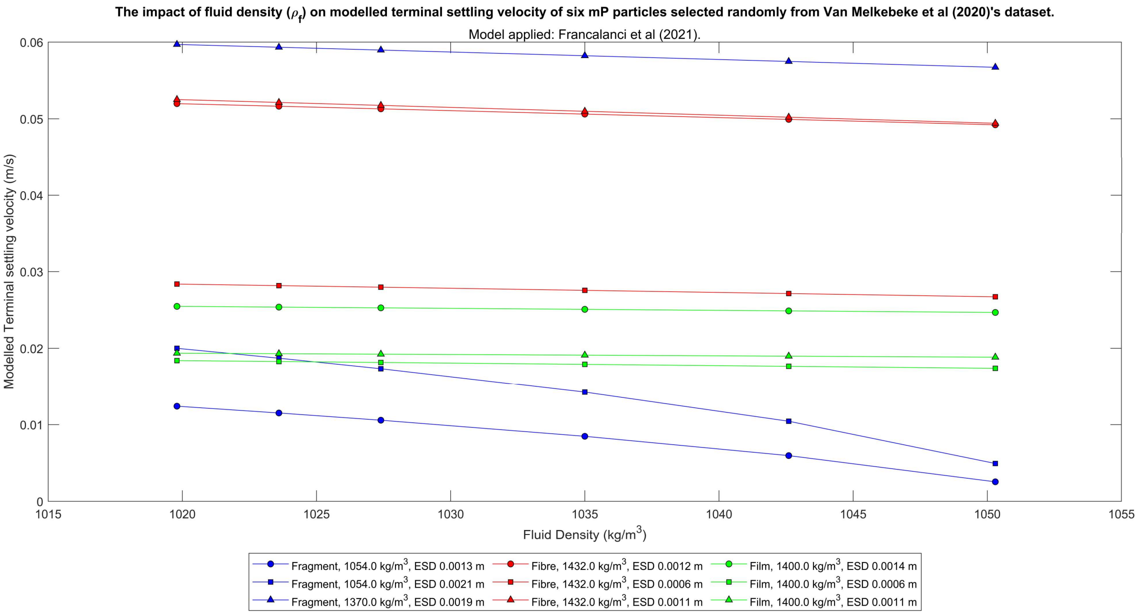

Figure S35: The influence of fluid density on the terminal settling velocity of six random particles using Francalanci *et al's*[6] model.

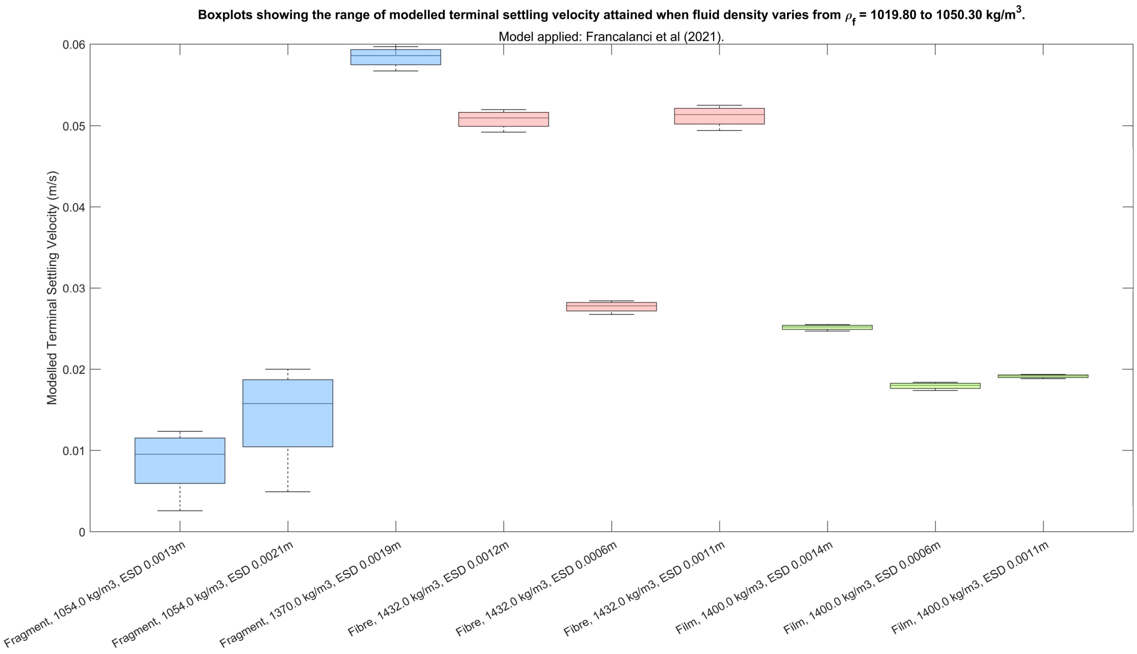

Figure S36: The range of settling velocity obtained for each of six random particles using Francalanci *et al's*[6] model when the fluid density was varied from 1019 to 1050 kg/m^3.

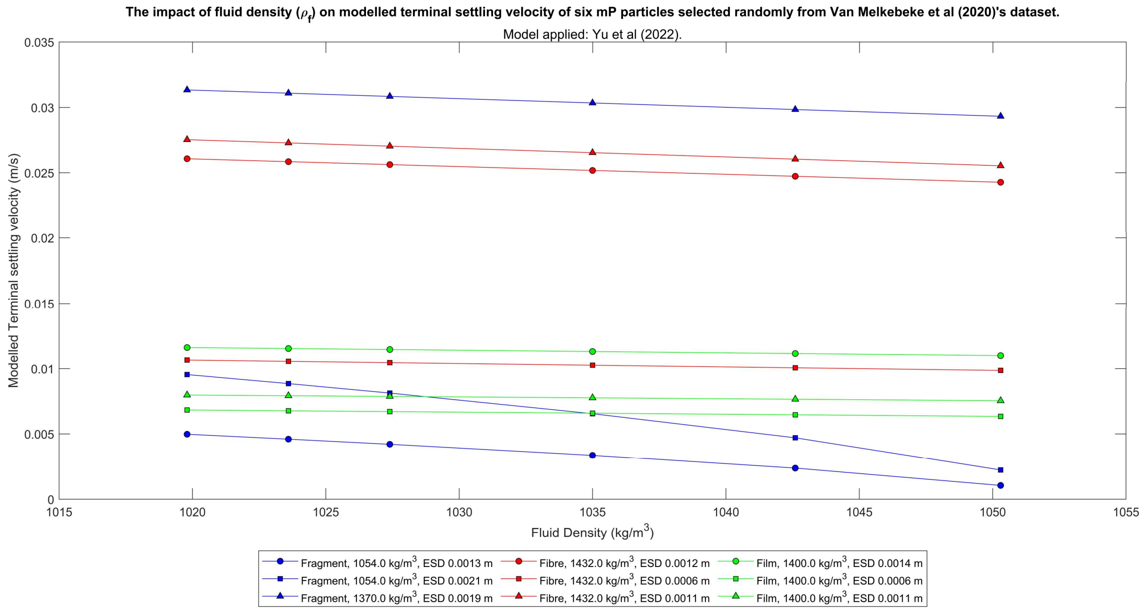

Figure S37: The influence of fluid density on the terminal settling velocity of six random particles using Yu*et al's*[7] model.

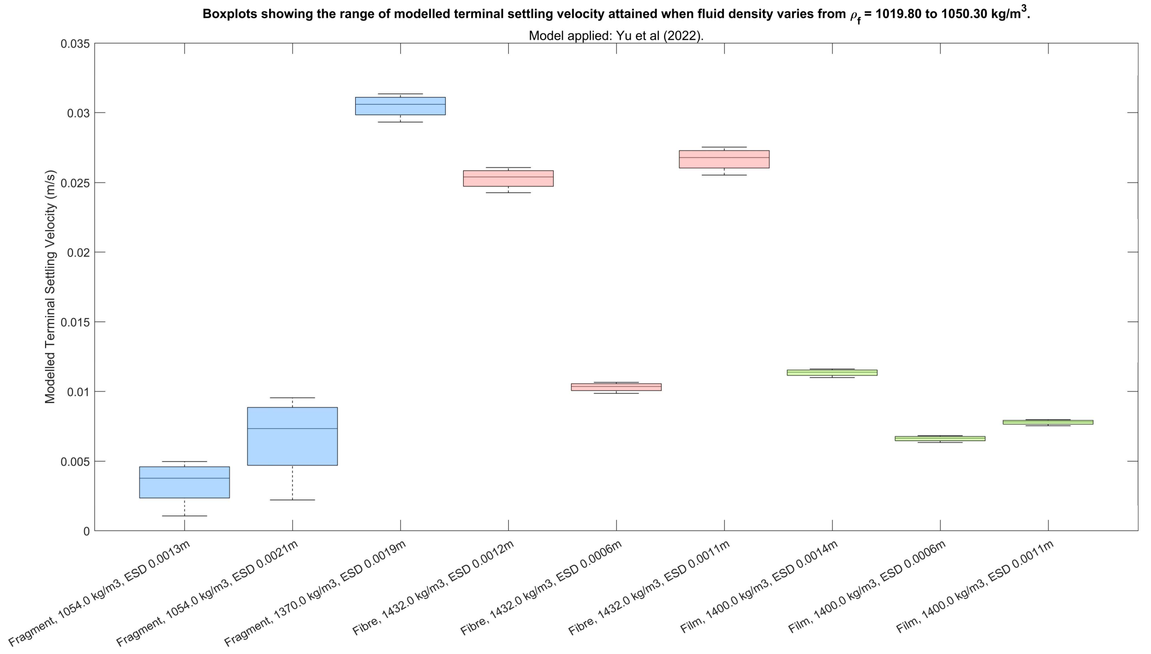

Figure S38: The range of settling velocity obtained for each of six random particles using Yu *et al's*[7] model when the fluid density was varied from 1019 to 1050 kg/m^3.

S1 13: Results for each model tested during the evaluation of the impact of using a constant terminal sinking velocity on the distance travelled by the mPs.

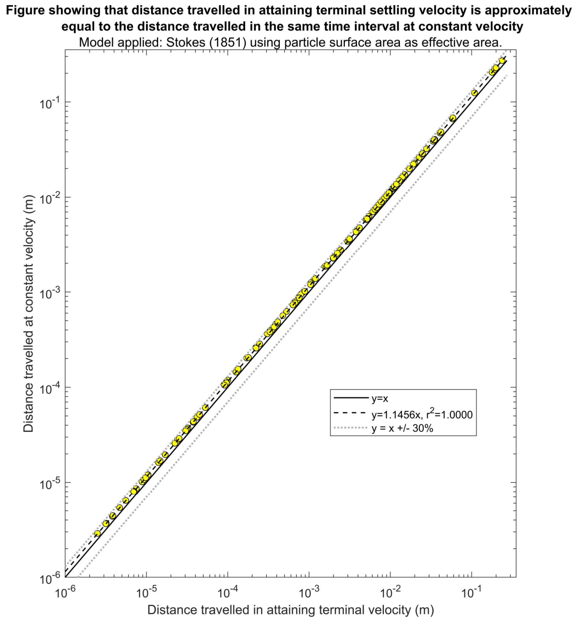

Figure S39: Comparison of the distance travelled in attaining the terminal settling velocity to the distance travelled if the particle sank constantly at the terminal settling velocity in the equivalent period of time when using Stokes model[1]. The solid line indicates the ideal fit where there is no difference in the distance travelled and the dotted lines indicate the distance travelled at a constant velocity is ±30% of the distance travelled whilst attaining terminal settling velocity. The dashed line indicates the best fit line in the form y=mx that was obtained using linear regression.

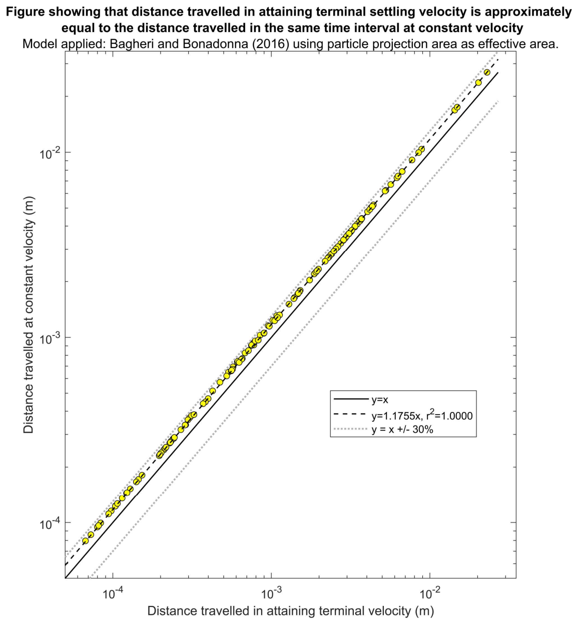

Figure S40: Comparison of the distance travelled in attaining the terminal settling velocity to the distance travelled if the particle sank constantly at the terminal settling velocity in the equivalent period of time when using Bagheri and Bonadonna's model[4]. The solid line indicates the ideal fit where there is no difference in the distance travelled and the dotted lines indicate the distance travelled at a constant velocity is ±30% of the distance travelled whilst attaining terminal settling velocity. The dashed line indicates the best fit line in the form y=mx that was obtained using linear regression.

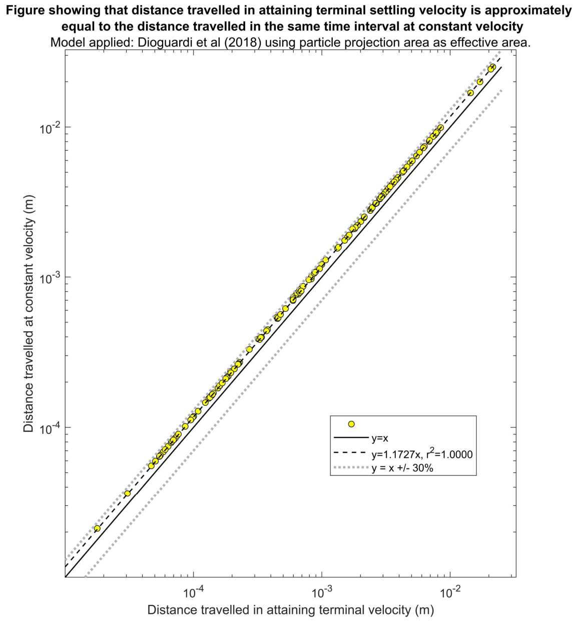

Figure S41: Comparison of the distance travelled in attaining the terminal settling velocity to the distance travelled if the particle sank constantly at the terminal settling velocity in the equivalent period of time when using Dioguardi *et al.*'s model[5]. The solid line indicates the ideal fit where there is no difference in the distance travelled and the dotted lines indicate the distance travelled at a constant velocity is ±30% of the distance travelled whilst attaining terminal settling velocity. The dashed line indicates the best fit line in the form y=mx that was obtained using linear regression.

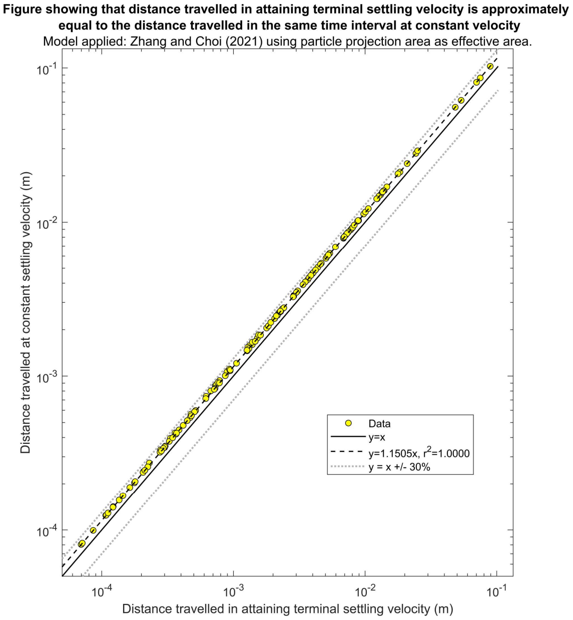

Figure S42: Comparison of the distance travelled in attaining the terminal settling velocity to the distance travelled if the particle sank constantly at the terminal settling velocity in the equivalent period of time when using Zhang and Choi's model[7] and taking the particle projected area as the effective area in the calculation of the drag force.The solid line indicates the ideal fit where there is no difference in the distance travelled and the dotted lines indicate the distance travelled at a constant velocity is ±30% of the distance travelled whilst attaining terminal settling velocity. The dashed line indicates the best fit line in the form y=mx that was obtained using linear regression.

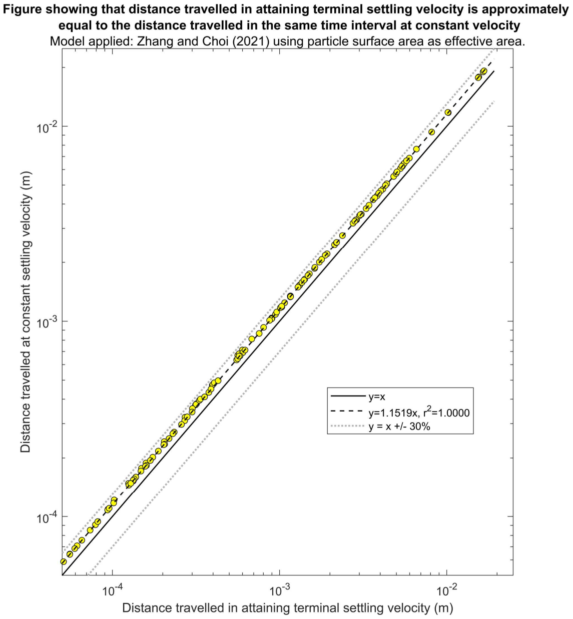

Figure S43: Comparison of the distance travelled in attaining the terminal settling velocity to the distance travelled if the particle sank constantly at the terminal settling velocity in the equivalent period of time when using Zhang and Choi's model[7] and taking the particle surface area as the effective area in the calculation of the drag force.The solid line indicates the ideal fit where there is no difference in the distance travelled and the dotted lines indicate the distance travelled at a constant velocity is ±30% of the distance travelled whilst attaining terminal settling velocity. The dashed line indicates the best fit line in the form y=mx that was obtained using linear regression.

Works cited:

1. Stokes, G. G., On the Effect of the Internal Friction of Fluids on the Motion of Pendulums. *Transactions of the Cambridge Philosophical Society* **1851,** *9*, 8.
2. Pye, K., *Sediment transport and depositional processes*. Blackwell Scientific Publications: 1994.
3. Gregory, J., *Particles in Water: Properties and Processes*. 1st ed ed.; CRC Press: 2005.
5. Bagheri, G.; Bonadonna, C., On the drag of freely falling non-spherical particles. *Powder Technology* **2016,** *301*, 526-544.
6. Dioguardi, F.; Mele, D.; Dellino, P., A New One-Equation Model of Fluid Drag for Irregularly Shaped Particles Valid Over a Wide Range of Reynolds Number. *Journal of Geophysical Research: Solid Earth* **2018,** *123* (1), 144-156.
7. Haider, A.; Levenspiel, O., Drag coefficient and terminal velocity of spherical and nonspherical particles. *Powder Technology* **1989,** *58* (1), 63-70.
8. Zhang, J.; Choi, C. E., Improved Settling Velocity for Microplastic Fibers: A New Shape-Dependent Drag Model. *Environmental Science & Technology* **2021**.
9. Van Melkebeke, M.; Janssen, C.; De Meester, S., Characteristics and Sinking Behavior of Typical Microplastics Including the Potential Effect of Biofouling: Implications for Remediation. *Environmental Science & Technology* **2020,** *54* (14), 8668-8680.
10. Dietrich, W. E., Settling velocity of natural particles. *Water Resources Research* **1982,** *18* (6), 1615-1626.
11. Francalanci, S.; Paris, E.; Solari, L., On the prediction of settling velocity for plastic particles of different shapes. *Environmental Pollution* **2021**, 118068.
12. Yu, Z.; Yang, G.; Zhang, W., A new model for the terminal settling velocity of microplastics. *Marine Pollution Bulletin* **2022,** *176*, 113449.
13. Dellino, P.; Mele, D.; Bonasia, R.; Braia, G.; La Volpe, L.; Sulpizio, R., The analysis of the influence of pumice shape on its terminal velocity. *Geophysical Research Letters* **2005,** *32* (21).
